# Improving $B_{1}^{+}$‐inhomogeneity tolerance by resolving non‐bijection in MP2RAGE *R*_1_ mapping: A 2D look‐up table approach demonstrated at 3 T

**DOI:** 10.1002/mrm.30363

**Published:** 2024-11-11

**Authors:** Lenno Ruijters, Torben Ellegaard Lund, Mads Sloth Vinding

**Affiliations:** ^1^ Center of Functionally Integrative Neuroscience, Department of Clinical Medicine Aarhus University Aarhus Denmark

**Keywords:** 2D look‐up table, B1+ inhomogeneity, MP2RAGE, non‐bijective data, *R*_1_‐mapping

## Abstract

**Purpose:**

Regarding in vivo, robust longitudinal relaxation rate (*R*_1_) mapping, the goal of the present paper is two‐fold. First, to verify that non‐bijective mapping in magnetization‐prepared 2 rapid gradient echo (MP2RAGE) imaging can be resolved through a two‐dimensional look‐up table approach. Second, that the expanded parameter space from this can be used to improve $B_{1}^{+}$‐inhomogeneity tolerance without other prerequisites.

**Theory:**

By deriving a second contrast from the magnitude images of the MP2RAGE acquisition, ambiguities in the original MP2RAGE image resulting from non‐bijective transfer curves can be resolved. Such ambiguities may occur when protocols are optimized, such as for higher $B_{1}^{+}$‐inhomogeneity tolerance. A 2D look‐up table approach combines the available information to resolve these ambiguities during mapping.

**Methods:**

At 3 T, we acquired MP2RAGE images with standard acquisition parameters and (non‐bijective) parameters optimized for $B_{1}^{+}$‐inhomogeneity tolerance. From 3 subjects across multiple sessions, we assessed the $B_{1}^{+}$‐inhomogeneity tolerance through excitation‐pulse amplitude scalings.

**Results:**

The *R*_1_ maps resulting from the $B_{1}^{+}$‐optimized protocols showed greatly reduced $B_{1}^{+}$ effects across images but without additional scanner time. Meanwhile, these maps could only successfully be derived by a 2D look‐up table approach.

**Conclusion:**

We show that it is possible to optimize for $B_{1}^{+}$‐inhomogeneity tolerance in MP2RAGE through sequence‐parameter settings, while still successfully estimating the *R*_1_ map with a two‐dimensional look‐up table approach. This without the need for an additional $B_{1}^{+}$ map. The increased parameter space enabled by the two‐dimensional look‐up table approach may further be used to adjust MP2RAGE acquisitions for improved scan times, signal‐to‐noise ratio, and/or contrast‐to‐noise ratio.

## 1 INTRODUCTION

In order for longitudinal relaxation rate (*R*_1_) mapping to achieve subject‐specific, in vivo, detailed maps of human cortical structures (building on Refs. 1, 2, 3), it is important that *R*_1_ changes reflect variations in myelo‐architecture rather than contaminations from artifacts. To map structurally distinct cortical areas beyond primary sensory tissue^1^, ^2^, ^3^ (i.e., whole‐brain), highly refined images are required. To detect subtle variations, these images need to be detailed and reliable (i.e., have high resolution and minimal artifact contributions). One prominent source of artifacts is $B_{1}^{+}$ inhomogeneity, which modulates the flip angle (FA), and consequently the measured signal. Factors that affect the size of $B_{1}^{+}$ effects include *B*_0_, readout train length, and repetition time (*T*_R_). The result is that, as higher resolutions become possible, $B_{1}^{+}$ artifacts increasingly counteract the benefits of increased spatial detail. Methodological strategies are necessary to limit the loss in *R*_1_ specificity as increased resolutions are achieved.

Magnetization‐prepared 2 rapid gradient echo (MP2RAGE) is a *T*_1_‐weighted MRI sequence that produces quantitative maps, while designed to mitigate *B*_1_ effects.^4^ By acquiring two rapid gradient echoes, and by combining these during postprocessing into a unified MP2RAGE image, $T_{2}^{*}$, *B*_0_, and *B*_1_^−^ effects can be eliminated. Calibrating sequence parameters, like the FAs or inversion times, can further minimize the $B_{1}^{+}$ effects, thereby reducing the need for $B_{1}^{+}$ map–based correction during postprocessing.^5^ Avoiding additional acquisition and correction steps minimizes scan times and accumulation of errors. Novel parallel‐transmit techniques additionally address $B_{1}^{+}$ effects during acquisition,^6^, ^7^, ^8^, ^9^, ^10^ although accessible implementations leave residual $B_{1}^{+}$ inhomogeneities.^9^ In certain situations, such as with increased number of slices, these residual inhomogeneities will leave severe bias in the resulting *R*_1_ maps.

Because MP2RAGE images are quantitative, the image values can be derived from *T*_1_ and the sequence parameters via Bloch simulations. If the mapping is bijective (i.e., one‐to‐one), then each MP2RAGE value can be uniquely associated with a *T*_1_ value.^11^ However, parameters with desirable features, including improved tolerance to $B_{1}^{+}$ inhomogeneities, high within‐tissue or between‐tissue contrast, or faster scanning times, may produce mappings that are non‐bijective. Consequently, *R*_1_ values are misestimated, and a different strategy is required to successfully derive *R*_1_ maps.

In this paper, we investigate the possibility of mitigating $B_{1}^{+}$ effects with modified sequence parameters by tackling the challenge of non‐bijective mappings between MP2RAGE values and *R*_1_. To this end, we introduce a new strategy able to derive *R*_1_ maps from MP2RAGE acquisitions with non‐bijective mappings. The present *R*_1_ mapping uses a secondary contrast, derived from the MP2RAGE acquisition, based on the ratio between the difference and the sum of the magnitude of the two gradient‐echo images (difference‐sum‐ratio [DSR]). Similar to the MP2RAGE image, effects related to $T_{2}^{*}$, *B*_0_, and *B*_1_^−^ are canceled out in the DSR image. Additionally, the DSR contrast is suitable for disambiguation of MP2RAGE values. Consequently, accurate *R*_1_ maps can be determined for non‐bijective mappings, as may be the case in protocols highly tolerant of $B_{1}^{+}$ inhomogeneity.

## 2 THEORY

Quantitative *T*_1_‐weighted images are derived from the MP2RAGE acquisition by combining the gradient‐echo images ($I_{1},I_{2}$) acquired from each inversion time ($T_{I,1},T_{I,2}$), voxel by voxel, into a unified image (*I*_UNI_), as follows:

(1) $$I_{\mathrm{UNI}}=R\left(\frac{I_{1}^{*}\cdot I_{2}}{{\left|I_{1}\right|}^{2}+{\left|I_{2}\right|}^{2}}\right)$$

**FIGURE 1:**
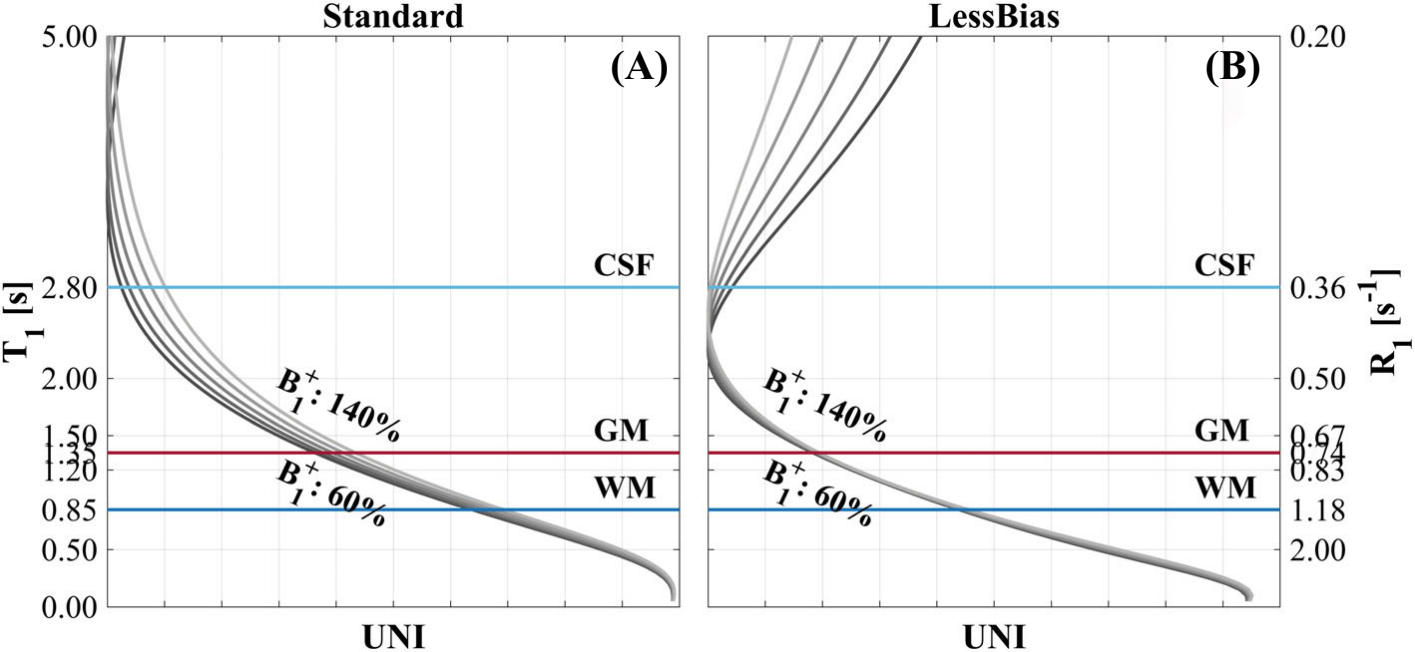
The magnetization‐prepared rapid gradient echo–to‐*T*_1_ transfer curves for the Standard (A) and LessBias (B) protocols, with corresponding *R*_1_ values on the right axis. The Standard protocol is more sensitive to $B_{1}^{+}$ inhomogeneities than the LessBias protocol, which is almost insensitive to $B_{1}^{+}$ variations in the order of ±40%. Within the range of *T*_1_ values from 0 s to 5 s, the Standard protocol has a near‐bijective relationship between the MP2RAGE uniform‐image (UNI) values and *T*_1_; this is not the case for the LessBias protocol, where within the *T*_1_ window, there are ambiguous UNI values. Also notice, we maintain a near identical contrast between gray matter (GM) and white matter (WM). CSF, cerebrospinal fluid.

When nuisance factors contribute equally to *I*_1_ and *I*_2_, these effects cancel out in *I*_UNI_. By nature of Eq. (1), *I*_UNI_ values are bound between −0.5 and + 0.5, and exactly equal to ±0.5, when *I*_1_ = ±*I*_2_. Because these values are quantitative, *I*_UNI_ values can be derived from *T*_1_ or *R*_1_(=1/*T*_1_). In standard implementations, *I*_UNI_ is mapped to *R*_1_ values via a one‐dimensional look‐up table (1D‐LUT), which can be visualized by a transfer curve (see Figure 1). A bijective relationship between *I*_UNI_ and *R*_1_ is required for the 1D‐LUT procedure to be applicable. This is achieved by careful selection of sequence parameters (Figure 1A).^4^ By scaling the FAs, the effects of $B_{1}^{+}$ inhomogeneity on estimated *R*_1_ values can be derived and visualized as separate transfer curves.

It is possible to select sequence parameters in which the corresponding transfer curve results in less dependency on $B_{1}^{+}$ variations (Figure 1B). However, such parameters may lead to non‐bijective mapping within the *R*_1_ window of interest. Here, the 1D‐LUT method fails, because *R*_1_ values from two different tissue types (e.g., gray matter [GM] and cerebrospinal fluid [CSF]) map to the same *I*_UNI_ value. These non‐bijections will occur when *I*_1_ = ±*I*_2_ at any point within the *R*_1_ window of interest. For standard protocols, non‐bijection will typically occur for low *R*_1_s (⪅ 0.2 s^−1^), leading to large *R*_1_ variance within CSF, which is normally unproblematic (however, see Section 5). For mappings in which the non‐bijection occurs at relatively high *R*_1_ values (like in Figure 1B), a solution is needed (see Supporting Information Section 3).

By introducing a second, distinct contrast that is derived from *I*_1_ and *I*_2_, we are able to solve ambiguities in non‐bijective mappings and accurately estimate the *R*_1_ values. This second contrast can be defined in several ways. We settled on a combination of the magnitudes of *I*_1_ and *I*_2_, as these magnitude images are readily available in standard setups. Similar to *I*_UNI_, we further wanted this contrast to be quantitative and tolerant to magnetic field inhomogeneities. The ratio between the difference of the first and second magnitude values and the sum (*I*_DSR_) satisfies these requirements, as follows:

(2) $$I_{\mathrm{DSR}}=\frac{|I_{1}|-|I_{2}|}{2\left(\;|I_{1}|+|I_{2}|\right)}$$

Multiplying the denominator by 2 ensures that *I*_DSR_ values fall within the same range as the *I*_UNI_ values. As *I*_DSR_ equals ±0.5 when either |*I*_1_| or |*I*_2_| is 0, and *I*_DSR_ equals 0 where |*I*_1_| = |*I*_2_|, the *I*_DSR_ contrast is indeed distinct from *I*_UNI_, where this behavior is inverted. Similar to *I*_UNI_, nuisance factors contributing equally to |*I*_1_| and |*I*_2_| are canceled out in *I*_DSR_.

**FIGURE 2:**
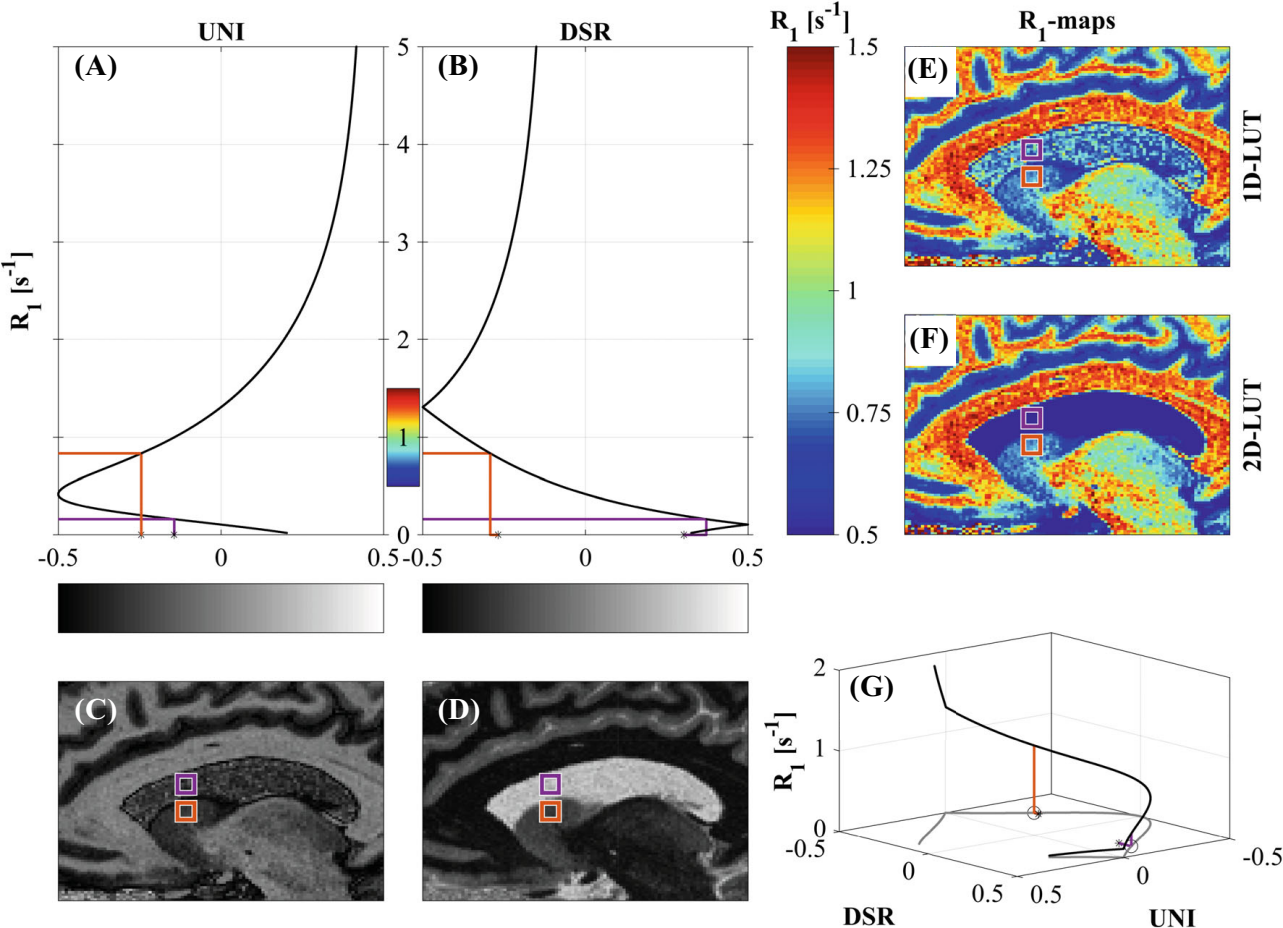
(A,B) One‐dimensional (1D) transfer curves for the magnetization‐prepared rapid gradient echo (MP2RAGE) uniform image (UNI; C) and difference‐sum‐ratio (DSR; D) of the LessBias protocol, respectively. (E,F) Resulting 1D and two‐dimensional (2D) lookup table (LUT) *R*_1_ maps, respectively. (G) The 2D transfer curve among MP2RAGE UNI, DSR, and *R*_1_. The MP2RAGE DSR (D) can be used to disentangle the ambiguous relationship between the MP2RAGE UNI image (C) and the *R*_1_ value (A, B). The method is illustrated for cerebrospinal fluid (CSF; *purple*) and deep gray‐matter (GM; *orange*) voxels with MP2RAGE UNI values close to each other. By Identifying the MP2RAGE values in both the MP2RAGE UNI image and the MP2RAGE DSR image, a unique *R*_1_ value can be assigned to each voxel using a 2D‐LUT (F) but not a 1D‐LUT (E). (G) The mapping is unambiguous in the full MP2RAGE UNI, DSR, and *R*_1_ space.

Successful non‐bijective mapping is performed by using a two‐dimensional lookup table (2D‐LUT), connecting a pair of (*I*_UNI_, *I*_DSR_) values to their corresponding *R*_1_ value, henceforth referred to as 2D‐LUT‐MP2RAGE. In its simplest implementation, the 2D‐LUT procedure compares the Euclidean distance between measured and reference value pairs to obtain the *R*_1_ value (here pdist2 in *MATLAB*; The MathWorks, Inc., Natick, MA, USA); this is illustrated in Figure 2. To ensure that the *R*_1_ estimation is driven by *I*_UNI_ values, as is the case for 1D‐LUT, a larger weight can be applied to *I*_UNI_ by equally scaling the measured and reference *I*_UNI_ values.

## 3 METHODS

The purpose of the present study is two‐fold: first, to verify that *R*_1_ mapping can be performed successfully in expanded sequence parameter space by resolving non‐bijective mapping through 2D‐LUT; and second, that from this expanded parameter space, parameters can be selected that result in *R*_1_ maps unaffected by $B_{1}^{+}$ inhomogeneities to an extent unseen without non‐bijective mapping. To demonstrate our proposed method, we acquired experimental data in a total of seven session across 3 subjects (the authors; see Table S1) on a 3T PrismaFit system (Siemens Healthineers, Erlangen, Germany) using a 32‐channel receive‐only head coil. Images were acquired with the default configuration of the host site (Standard) and a new protocol (LessBias) with improved tolerance to $B_{1}^{+}$ inhomogeneities in the final *R*_1_ maps: (1) Standard: *T*_R__,MP2RAGE_ = 5 s, *T*_R__,FLASH_ = 7.18 ms, FA_1_/FA_2_ = 4°/5°, *T*_I,1_/*T*_I,2_ = 700 ms/2500 ms; (2) LessBias: *T*_R__,MP2RAGE_ = 5 s, *T*_R__,FLASH_ = 7.18 ms, FA_1_/FA_2_ = 3°/5°, *T*_I,1_/*T*_I,2_ = 500 ms/1900 ms. In both cases, a single acquisition took 11:20 min with a 215.6 × 172.8 × 230 mm^3^ field of view (anterior–posterior/left–right/superior–inferior; phase/slice/read), grid size = 256 × 192 × 240 (i.e., 0.9‐mm^3^ isotropic voxels, partial Fourier = 6/8, and 2xGRAPPA).^12^ FatNavs motion correction was applied.^13^, ^14^ To systematically modify $B_{1}^{+}$ variation, we modified the excitation pulse amplitude (“SRFExcit” in the scan card; Figure S1), which is equivalent to using noninteger FAs. Each protocol was acquired 3 times, with SRFExcit at 60%, 100%, and 140% (i.e., FA_1_/FA_2_ = 2.4°/3° for Standard‐60%) of the reference voltage. This range reflects the $B_{1}^{+}$ inhomogeneities seen beyond 3 T, allowing us to get an indication on how it would fare at higher field strengths, such as at 7 T,^4^ and fully stress‐test the 2D‐LUT‐MP2RAGE at 3 T.

Following FatNavs motion correction,^13^, ^14^ the anterior and posterior commissure were identified on the Standard 100% excitation‐pulse amplitude image. Next, all other images were reoriented accordingly, to obtain a near–anterior and posterior commissure space orientation in all acquisitions. For each acquisition, an *R*_1_ map was created using 1D‐LUT^11^ and 2D‐LUT for Standard and LessBias acquisitions, respectively.

The 2D‐LUT *R*_1_ mapping was performed using a step size of Δ*R*_1_ = 0.001 s^−1^ from *R*_1_ = 0.01 s^−1^ to 20 s^−1^. These hyperparameters were chosen empirically to avoid 2D‐LUT truncation effects. We opted for equidistant *R*_1_ spacing over equidistant *T*_1_ spacing, to better capture variations in the WM‐GM range. To compare 1D‐LUT and 2D‐LUT *R*_1_ mapping most directly, we applied a strong, empirically determined 100:1 weight ratio between *I*_UNI_ and *I*_DSR_ before calculating the Euclidian distance. Consequently, *R*_1_ values were assigned on the basis of the *I*_UNI_ values, with *I*_DSR_ values used primarily for disambiguation.

The resultant *R*_1_ maps were segmented using *SPM12* r7711,^15^, ^16^ and from the segmented images, two masked *R*_1_ maps were created. The first masked image was constructed from a one‐step eroded brain mask containing voxels where the sum of posterior probabilities for CSF, GM, and WM exceeded 0.2. The second was constructed similarly, but included skull and soft tissue voxels. The brain‐only *R*_1_ maps were coregistered to the Standard 100% excitation‐pulse amplitude acquisition, and the estimated registrations were applied to all images. From these brain‐masked, coregistered *R*_1_ maps, mean and standard deviation maps across the different excitation‐pulse amplitude scalings for both protocols (Standard and LessBias) were calculated. These mean and standard deviation maps were used to assess the standard deviation within each tissue type and visualize the $B_{1}^{+}$ effects in WM and CSF.

The effects within GM were visualized on a central GM surface. Central GM surfaces were constructed using *CAT12* r2560^17^, ^18^, ^19^, ^20^ to segment the head‐masked, coregistered *R*_1_ maps with the iso‐volume approach.^21^, ^22^ By sampling each *R*_1_ map on their corresponding central GM surface and projecting out the local curvature,^1^ we obtained curvature‐corrected central GM *R*_1_ surfaces for each image. Before curvature correction, both curvature and *R*_1_ surfaces were resampled to the *FreeSurfer* template surface in *CAT12*, and 2D‐smoothed with a Gaussian kernel (full width at half maximum = 6 mm). From these surfaces, we calculated the standard deviation across the different excitation‐pulse amplitude scalings, for both protocols. The curvature‐corrected *R*_1_ values and their standard deviations were then visualized on an inflated GM template surface.

To compare the effects of noise and $B_{1}^{+}$ inhomogeneity on *R*_1_, we acquired three images for both protocols in 1 subject with unmanipulated excitation pulses. Instead, the nominal FA provided during *R*_1_ mapping was manipulated in steps of 10%. Ten *R*_1_ maps were derived for each image, where the FA for one image was left untouched; the FA for one image was reduced by 0% to 90%; and the FA in the last image was increased by 0% to 90%. Preprocessing of these images was left as described previously.

In addition, we acquired SA2RAGE images during each session to compare the efficacy of LessBias against postprocessing $B_{1}^{+}$‐map correction.^5^ $B_{1}^{+}$ correction was applied to the Standard images using in‐house code (see Supporting Information Section 7), and the corrected *R*_1_ maps were compared with both uncorrected Standard and LessBias *R*_1_ maps (see Supporting Figures S11–S15).

## 4 RESULTS

The signal‐to‐noise ratio (SNR) between Standard and LessBias is highly comparable for nominal excitation FAs (i.e., SRFExcit of 100%). When SNR is defined as the maximum intensity value across |*I*_1_| and |*I*_2_| divided by the mean intensity value of the background of |*I*_1_|, SNR_Standard_ ≈ 75 and SNR_LessBias_ ≈ 71.

Figure 1 shows the transfer curves of Standard (1A) and LessBias (1B), the latter showing non‐bijection within the *R*_1_ window of interest (0.2–5 s^−1^), with the fold occurring at approximately *R*_1_ = 0.4 s^−1^. Additionally, the estimated $B_{1}^{+}$ inhomogeneity tolerance in the GM and WM range for LessBias is strongly improved compared with Standard. Figure 2 visualizes the 2D‐LUT‐MP2RAGE. Figure 2E shows how the 1D‐LUT approach gives rise to ambiguous *R*_1_ values across CSF (purple square) and GM (orange square). Figure 2F shows how 2D‐LUT resolves the non‐bijective mapping.

**FIGURE 3:**
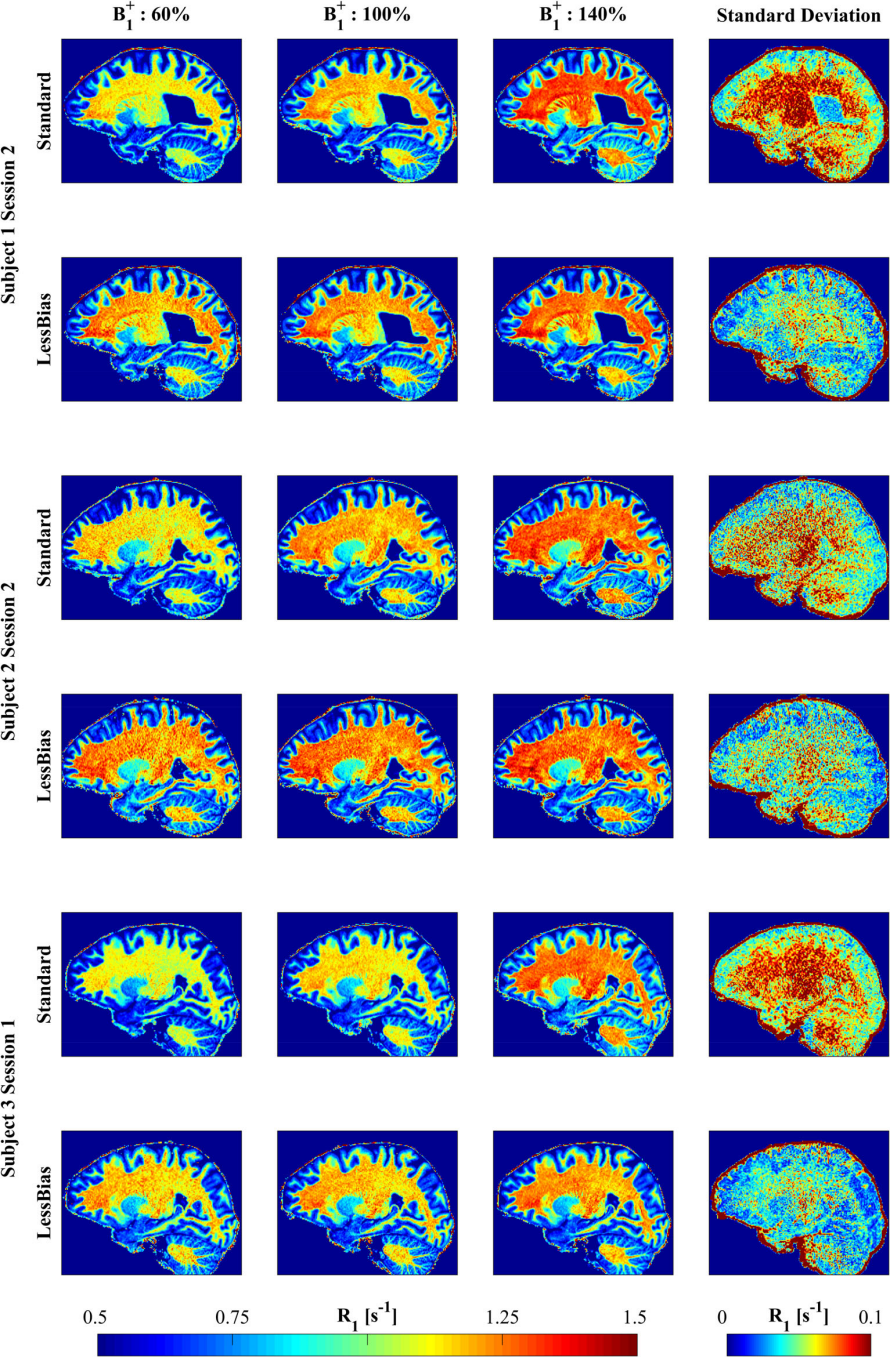
Volumetric *R*_1_ maps from the Standard (*odd rows*) and LessBias (*even rows*) protocol for Subject 1 (*top*), 2 (*middle*), and 3 (*bottom*). By scaling the excitation pulse amplitude to 60%, 100%, and 140% (*Columns 1–3, respectively*), it is possible to visualize the benefit of the LessBias protocol. As shown from both the individual acquisitions and the standard deviation image (*Column 4*), the LessBias protocol does indeed, as predicted from Figure 1, demonstrate robustness to deviations from the nominal $B_{1}^{+}$. From the standard deviation images, it can be observed that LessBias provides a strongly reduced standard deviation for gray matter and white matter, at the cost of a slightly increased standard deviation for cerebrospinal fluid, compared with the Standard protocol. Actual mean and standard deviation of *R*_1_ values are listed in Table S6.

**FIGURE 4:**
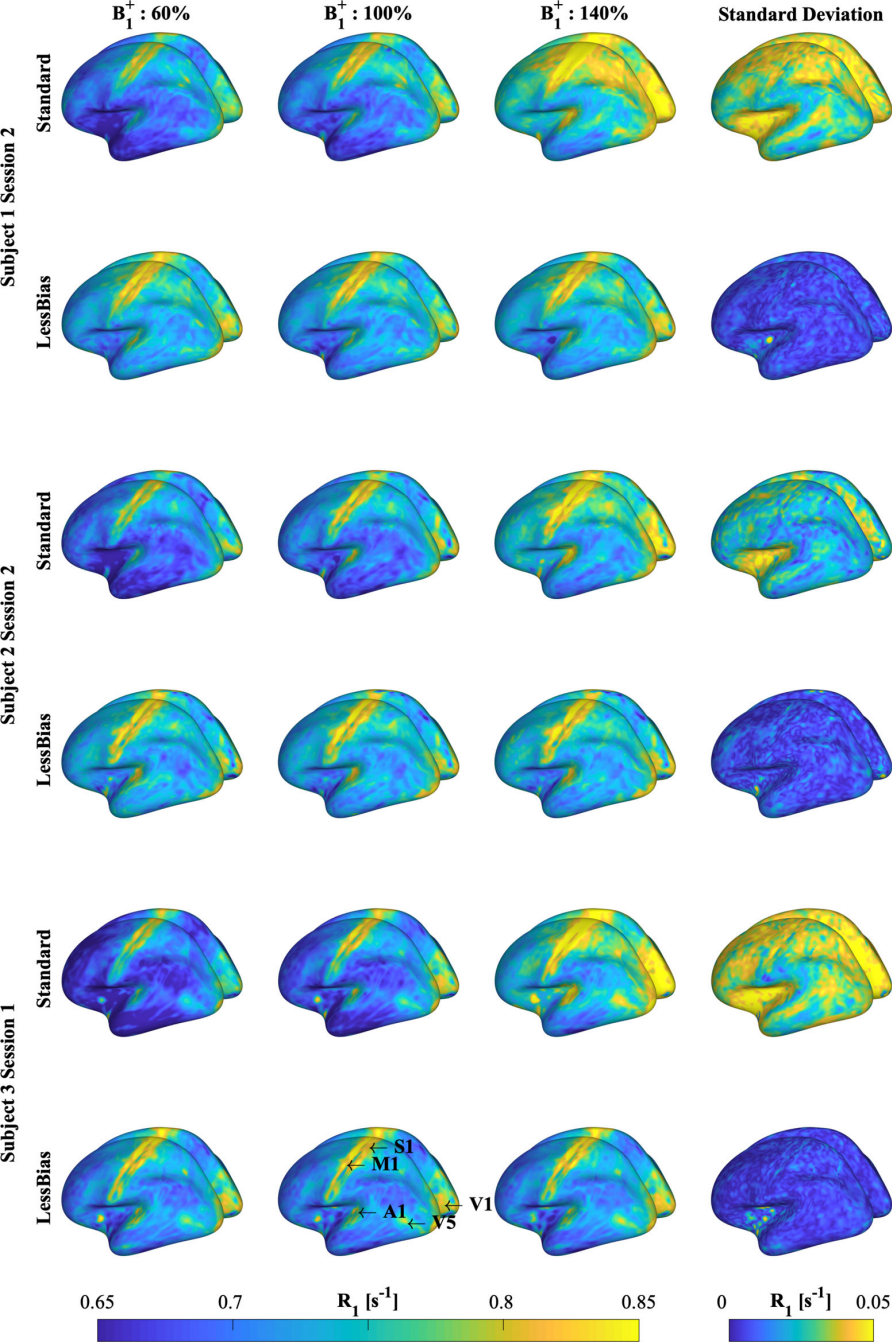
Gray‐matter (GM) surface *R*_1_ maps from the Standard (*odd rows*) and LessBias (*even rows*) protocol for Subject 1 (*top*), 2 (*middle*), and 3 (*bottom*). GM surfaces are presented with uniform windowing across images (0.65–0.85 s^−1^). By scaling the excitation pulse amplitude to 60%, 100%, and 140% (*Columns 1–3, respectively*), it is possible to visualize the benefit of the LessBias protocol. As shown from both the individual acquisitions and the standard deviation surface (*Column 4*), the LessBias protocol does indeed, as predicted from Figure 1, demonstrate robustness to deviations from the nominal $B_{1}^{+}$. From the standard deviation surfaces, it appears that LessBias provides a strongly reduced standard deviation for GM, compared with the Standard protocol. Increased *R*_1_ values are observed in expected regions (V1, M1, S1, A1, and possibly V5). Cortical GM volume and thickness estimations are listed in Table S3. Actual mean and standard deviation of *R*_1_ values are listed in Table S6.

Figures 3 and 4 outline the results of amplifying $B_{1}^{+}$ across acquisitions. In Figure 3, substantial WM *R*_1_ variation is seen across the different levels of $B_{1}^{+}$ amplification in Standard for Subjects 1–3. In contrast, LessBias combined with 2D‐LUT provide consistent WM *R*_1_ values across $B_{1}^{+}$ amplifications. For a detailed overview of the standard deviations across protocols, see Tables S5–S7.

In Figure 4, the variability in *R*_1_ values is displayed on central GM surfaces across Subjects 1–3. Standard (odd rows) shows substantial variation in *R*_1_ values compared with LessBias (even rows). This is further illustrated by the standard deviation across each row (final column), where the standard deviations for LessBias are consistently substantially lower than for Standard. Highly myelinated areas (e.g., V1/V5‐MT+/M1/S1/A1^1^, ^2^, ^3^) are reliably identifiable across LessBias images, whereas they become less distinct in Standard (e.g., V1 in Subject 1–Standard 140% or Subject 3–Standard 60%).

**FIGURE 5:**
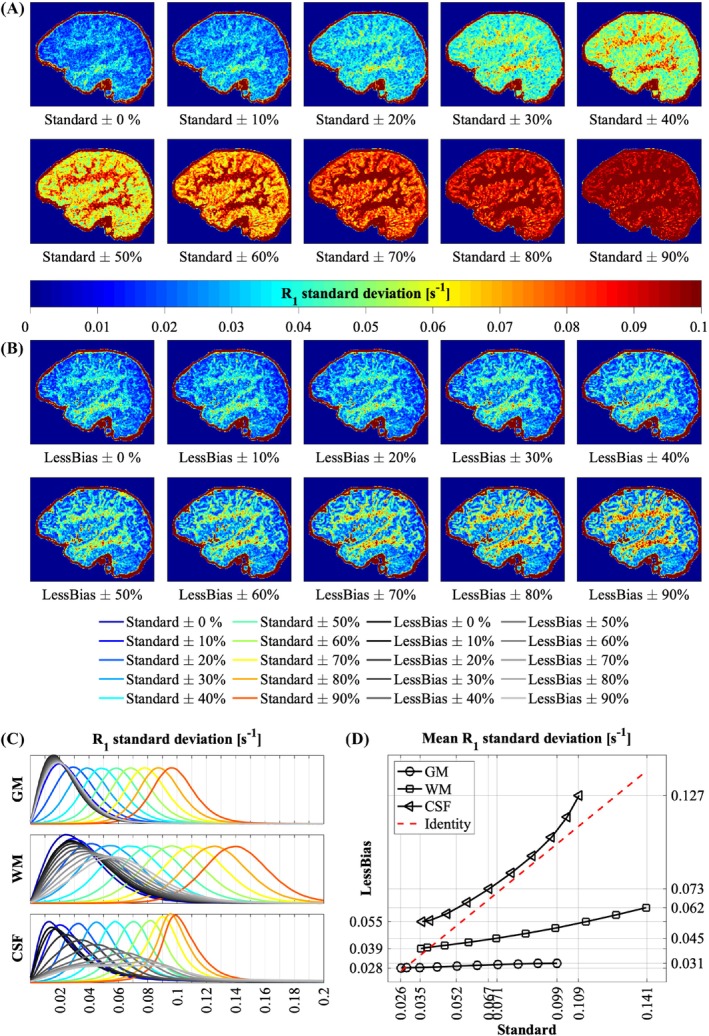
(A) *R*_1_ standard deviation maps of Standard for flip‐angle (FA) manipulations performed during *R*_1_ map estimation. (B) *R*_1_ standard deviation maps of LessBias for FA manipulations performed during *R*_1_‐map estimation. (C) Distributions of voxel‐wise *R*_1_ standard deviations per manipulation for Standard and LessBias (*in color and grayscale, respectively*). For each tissue type (gray matter [GM], white matter [WM], and cerebrospinal fluid [CSF]), a mask was created from the intersections between Standard and LessBias of the segmented volumes from the unmanipulated ($B_{1}^{+}$: 100%) Standard and LessBias images. (D) Mean of the standard deviations distributions of (C) for GM, WM, and CSF plotted against each other per manipulation. The leftmost/rightmost points belong to 0% and 90% manipulation, respectively. We expect that variation seen in (A) and (B) at ±0% is primarily driven by noise. Variation introduced as a result of the FA manipulation is assumed to be analogue to bias‐induced variation. Visually, it appears in (A) that bias overtakes as the driving factor for variation at ±20%. This is confirmed by the histograms in (C), where especially in GM and WM, the distribution of standard deviations appears to be a linear shift based on the FA manipulation. This becomes more apparent where we see that the contribution of an FA manipulation of 20% and above greatly outweighs the contribution of a ±10% manipulation. Meanwhile, visual variation in LessBias for WM and especially GM remains minimal for a far higher FA manipulation, as seen in (B). Distribution of standard deviations as a result of the manipulation in (C) is far less spread out in GM and WM for LessBias than Standard. In (D), we see that the mean standard deviation in GM is increased by about 10% at a ±90% FA manipulation, and in WM about 20% at a ±40% FA manipulation (vs. ˜300% and ˜75% in Standard respectively).

Figure 5 shows the results of manipulating the nominal FA during *R*_1_ mapping across comparable images from one session. At an FA manipulation of ±0%, we expect the variance across images to be driven primarily by noise. Differences in standard deviation at ±0% and the FA manipulations indicate the contribution of bias.

The Supporting Information (overview in Table S1) further describes quantitative *R*_1_ accuracy assessment with respect to curvature correction (Section 4), segmentation robustness (Section 5), mean *R*_1_ values across subjects, protocols and sessions (Section 6), FatNavs effects (Section 6), and influence of number of slices (Sections 5 and 6).

## 5 DISCUSSION

With 2D‐LUT‐MP2RAGE *R*_1_ mapping, we have expanded the MP2RAGE sequence parameter space, enabling us to further refine the features of MP2RAGE acquisitions. In this paper, we focused on the potential for improving $B_{1}^{+}$‐inhomogeneity tolerance on the path toward ultrahigh resolution. Given the push for higher field strengths,^23^, ^24^ driven by this desire for higher resolutions, the problem of $B_{1}^{+}$‐inhomogeneity intolerance will only become more pressing. To investigate levels of $B_{1}^{+}$ inhomogeneities similar to those seen at 7 T, we manipulated the amplitude of the excitation pulse across images. It appears indeed possible to achieve greatly improved $B_{1}^{+}$‐inhomogeneity tolerance with minimal SNR compromise. Using the adjusted sequence parameters in combination with 2D‐LUT mapping, we drastically reduced the standard deviation of WM and GM tissue volumes (see Tables S2–S4) and *R*_1_ values (see Tables S5–S7) without extending the acquisition time. This further indicates that differences across manipulations are smaller for LessBias than Standard, confirming the former is more robust against $B_{1}^{+}$ effects.

Figure 5 shows that, for the Standard protocol, $B_{1}^{+}$ contributions to variance compared with noise are already substantial at ±20%. Meanwhile, Figure 5 suggests that LessBias is far less susceptible to $B_{1}^{+}$effects in GM and WM, showing nearly no effect of bias in GM and a drastically reduced effect in WM compared with Standard. Similarly, variability in CSF is greatly reduced in LessBias up to a point, showing a shift at about 40%. This shift could be because the analysis captures consistency, not accuracy. Beyond a certain FA manipulation, variability may top out, slowing down the trajectory of Standard. It should also be noted that the SNR during each manipulation remains constant, whereas variations in the FA during acquisition would result in altered SNRs. As a result, Figure 5 cannot catch the total effect of $B_{1}^{+}$ inhomogeneities, but only the variation driven by deviations from the nominal transfer curve.

There are alternative methods that correct for $B_{1}^{+}$ either in postprocessing^25^, ^26^ (e.g., SA2RAGE^5^ or MP3RAGE^27^) or at the acquisition stage, (e.g., parallel transmission^6^, ^7^ and universal pulses).^8^, ^9^, ^10^ For example, parallel‐transmit PASTeUR universal pulses substantially reduced $B_{1}^{+}$ inhomogeneities at 7 T compared with circular polarization mode.^9^ The remaining $B_{1}^{+}$ inhomogeneity should be manageable with 2D‐LUT‐MP2RAGE. Note that the parameters used in the protocol can be adjusted to the user's needs but at the expense of other parameters. For instance, the number of slices can be increased for higher resolution imaging, but maintaining $B_{1}^{+}$‐inhomogeneity tolerance requires additional adjusting of inversion times and FAs. The extent of these adjustments would further depend on what other $B_{1}^{+}$ inhomogeneity–compensation techniques are available and the *B*_0_ (for an example, see Supporting Information Section 8).

Whether or not MP2RAGE is acquired in combination with, for example, universal pulses, we see no immediate downsides of using 2D‐LUT‐MP2RAGE, given that the potential benefits come at virtually no cost compared with standard MP2RAGE applications.

LessBias, made possible with 2D‐LUT‐MP2RAGE, provides $B_{1}^{+}$‐inhomogeneity tolerance at the voxel level without modified sequences or increased scan times, and with sufficient time for fat navigators. Time otherwise needed for such high‐resolution $B_{1}^{+}$‐inhomogeneity corrections can instead be used to improve throughput, SNR, or resolution of *R*_1_ images. Given that the LessBias protocol is agnostic to hardware setup and availability of secondary sequences, we anticipate that it can be implemented to tackle the challenges of cross‐site collaborations^28^ to a larger extent than alternative methods.

Recent developments with synthetic *T*_1_ contrasts showed that through a sufficiently accurate *T*_1_ map, different *T*_1_‐weighted contrasts can be derived in postprocessing, and that this can be used to optimize scan times.^8^ When relying on synthetic MRI, the contrast of the original acquisition can be arbitrary, as long as the *T*_1_ mapping succeeds. This means that *I*_UNI_ no longer needs to be visually interpretable and permits non‐bijection. Consequently, further optimizations for improved $B_{1}^{+}$‐inhomogeneity tolerance, SNR, and/or acquisition times may be possible in the wider sequence parameter space. Desired (visually interpretable) contrasts can then be computed from the resultant *T*_1_ maps.

Other implementations for non‐bijective transfer curves may be for specialized uses (e.g., Ref. 29). Such cases may require sequence parameters that come with increased contrast‐to‐noise ratio in or across specific tissues (e.g., simultaneous brain and cervical spinal cord imaging). Reducing the slope of the transfer curve will not only lead to increased contrast between tissues in *I*_UNI_ but will also lead to non‐bijective transfer curves when the slope drops below a certain threshold. When such protocols are desired, 2D‐LUT‐MP2RAGE will still be able to derive accurate *R*_1_ maps from the data. Through additional postprocessing steps, the high contrast from the original images could be extracted, whereas the *R*_1_ map serves as a reference.

The remaining CSF *T*_1_‐variance compromise impose limits such as in specific CSF‐based MRI biomarkers and neurodegenerative‐disease studies.^30^ However, we stress that Standard suffered from stronger variance than LessBias. Through 2D‐LUT‐MP2RAGE, it may be possible to select parameters that address *T*_1_ variance in CSF, but this was not investigated. Additionally, Standard suffers from non‐bijections around CSF at a low $B_{1}^{+}$, leading to larger misestimations in 1D‐LUT that cannot be corrected with a 1D‐LUT $B_{1}^{+}$‐correction method.

This study assumed 96% inversion efficiency,^4^ which anticipatedly is fairly valid at 3 T. There is evidence that at ultrahigh field strength this assumption is compromised,^27^ and investigations with respect to inversion efficiency and our method remain. This is where 2D‐LUT‐MP2RAGE may best be combined with universal pulses.

Our $B_{1}^{+}$ maps (see Figure S16) show an expected ±20% variation, but we stress‐tested our method with ±40% excitation‐pulse amplitude scalings to mimic variations seen commonly at 7 T^4^ as a feasibility test for future studies. This study does not directly answer how more severe variations are attacked, but as mentioned previously, we expect the method to add value in concert with other methods, such as using universal pulses.^9^, ^10^

## 6 CONCLUSIONS

With the 2D‐LUT‐MP2RAGE, we were able to open up the usable MP2RAGE acquisition parameter space, which can impart non‐bijective MP2RAGE‐to‐*T*_1_ transfer curves. This increased freedom was used to address $B_{1}^{+}$ inhomogeneity without additional hardware or data than what the current MP2RAGE sequence inherently provides. Compared with typical MP2RAGE acquisition parameters, we saw greatly reduced variability across GM and WM *R*_1_ values with $B_{1}^{+}$ variations (±40%). Although this paper focused on improving $B_{1}^{+}$‐inhomogeneity tolerance, this 2D‐LUT mapping may also enable the use of protocols that provide contrast or scan times previously unseen in MP2RAGE.

## Supporting information


**Data S1** Supporting information.

## Data Availability

Code and example data set are available at https://github.com/torbenelund/2D‐lookup‐tools‐for‐MP2RAGE.

## References

[1] Lutti A , Dick F , Sereno MI , Weiskopf N . Using high‐resolution quantitative mapping of R1 as an index of cortical myelination. Neuroimage. 2014;93:176‐188. doi:10.1016/j.neuroimage.2013.06.005 23756203

[2] Dick F , Taylor Tierney A , Lutti A , Josephs O , Sereno MI , Weiskopf N . In vivo functional and myeloarchitectonic mapping of human primary auditory areas. J Neurosci. 2012;32:16095‐16105. doi:10.1523/JNEUROSCI.1712-12.2012 23152594 PMC3531973

[3] Glasser MF , Van Essen DC . Mapping human cortical areas in vivo based on myelin content as revealed by T_1_‐ and T_2_‐weighted MRI. J Neurosci. 2011;31:11597‐11616. doi:10.1523/JNEUROSCI.2180-11.2011 21832190 PMC3167149

[4] Marques JP , Kober T , Krueger G , van der Zwaag W , Van de Moortele PF , Gruetter R . MP2RAGE, a self bias‐field corrected sequence for improved segmentation and T_1_‐mapping at high field. Neuroimage. 2010;49:1271‐1281. doi:10.1016/j.neuroimage.2009.10.002 19819338

[5] Eggenschwiler F , Kober T , Magill AW , Gruetter R , Marques JP . SA2RAGE: a new sequence for fast $B_{1}^{+}$ −mapping. Magn Reson Med. 2012;67:1609‐1619. doi:10.1002/mrm.23145 22135168

[6] Zhu Y . Parallel excitation with an array of transmit coils. Magn Reson Med. 2004;51:775‐784. doi:10.1002/mrm.20011 15065251

[7] Katscher U , Börnert P , Leussler C , van den Brink JS . Transmit SENSE. Magn Reson Med. 2003;49:144‐150. doi:10.1002/mrm.10353 12509830

[8] Bapst B , Massire A , Mauconduit F , et al. Pushing mp2rage boundaries: ultimate time‐efficient parameterization combined with exhaustive t _ 1 _ synthetic contrasts. Magn Reson Med. 2024;91:1608‐1624. doi:10.1002/mrm.29948 38102807

[9] Mauconduit F , Massire A , Gras V , Amadon A , Vignaud A , Boulant N . PASTeUR package extension with MP2RAGE for robust T_1_ mapping technique in parallel transmit at 7T. Proceeding of the 2020 ISMRM & SMRT Virtual Conference & Exhibition. International Society for Magnetic Resonance in Medicine and International Society for MR Radiographers & Technologists; 2020:3709.

[10] Mauconduit F , Diraison T , Massire A , et al. Pushing the image quality by integrating FatNav and pTx universal pulses in MPRAGE and MP2RAGE sequences at 7T. In: *Proceedings of the* 2024 ISMRM & SMRT Virtual Conference & Exhibition. International Society for Magnetic Resonance in Medicine and International Society for MR Radiographers & Technologists; 2024. p. 4895.

[11] Marques JP . MP2RAGE scripts. https://github.com/JosePMarques/MP2RAGE‐related‐scripts. Accessed April 5, 2024.

[12] Griswold MA , Jakob PM , Heidemann RM , et al. Generalized autocalibrating partially parallel acquisitions (GRAPPA). Magn Reson Med. 2002;47:1202‐1210. doi:10.1002/mrm.10171 12111967

[13] Gallichan D , Marques JP , Gruetter R . Retrospective correction of involuntary microscopic head movement using highly accelerated fat image navigators (3D FatNavs) at 7T. Magn Reson Med. 2016;75:1030‐1039. doi:10.1002/mrm.25670 25872755

[14] Gallichan D . Retro‐MoCo‐Box. https://github.com/dgallichan/retroMoCoBox. Accessed April 5, 2024.

[15] Friston K , Ashburner J , Kiebel S , Nichols T , Penny W , eds. Statistical Parametric Mapping. UK: Academic Press; 2007:625–647. doi:10.1016/B978-012372560-8/50050-4

[16] The University College London . *SPM* (Statistical Parametric Mapping). 2020. https://www.fil.ion.ucl.ac.uk/spm‐statistical‐parametric‐mapping/. Accessed April 5, 2024.

[17] Gaser C , Dahnke R , Thompson PM , Kurth F , Luders E . Alzheimer's disease neuroimaging initiative. CAT: a computational anatomy toolbox for the analysis of structural MRI data. GigaScience. 2024;13. doi:10.1093/gigascience/giae049 PMC11299546 39102518

[18] Yotter RA , Dahnke R , Thompson PM , Gaser C . Topological correction of brain surface meshes using spherical harmonics. Hum Brain Mapp. 2011;32:1109‐1124. doi:10.1002/hbm.21095 20665722 PMC6869946

[19] Dahnke R , Yotter RA , Gaser C . Cortical thickness and central surface estimation. Neuroimage. 2013;65:336‐348. doi:10.1016/j.neuroimage.2012.09.050 23041529

[20] Computational Anatomy Toolbox CAT12. https://neuro‐jena.github.io/cat12‐help/. Accessed April 8, 2024.

[21] Consolini J , Demirci N , Fulwider A , Hutsler JJ , Holland MA . Bok's equi‐volume principle: translation, historical context, and a modern perspective. Brain Multiphys. 2022;3:100057. doi:10.1016/j.brain.2022.100057

[22] Dinse J , Härtwich N , Waehnert MD , et al. A cytoarchitecture‐driven myelin model reveals area‐specific signatures in human primary and secondary areas using ultra‐high resolution in‐vivo brain MRI. Neuroimage. 2015;114:71‐87. doi:10.1016/j.neuroimage.2015.04.023 25896931

[23] NWO . The Dutch National 14 Tesla MRI Initiative in Medical Science (Dynamic). NWO. https://www.nwo.nl/en/projects/184036009. Accessed April 2, 2024.

[24] U.S. Food and Drug FDA clears first 7T magnetic resonance imaging device, administration. https://www.fda.gov/news‐events/press‐announcements/fda‐clears‐first‐7t‐magnetic‐resonance‐imaging‐device. Accessed April 2, 2024.

[25] Weiskopf N , Suckling J , Williams G , et al. Quantitative multi‐parameter mapping of R_1_, PD*, MT, and R2* at 3T: a multi‐center validation. Front Neurosci. 2013;7:95. doi:10.3389/fnins.2013.00095 23772204 PMC3677134

[26] Marques JP , Gruetter R . New developments and applications of the MP2RAGE sequence—focusing the contrast and high spatial resolution R1 mapping. Yacoub E, ed. PLoS One. 2013;8:e69294. doi:10.1371/journal.pone.0069294 23874936 PMC3712929

[27] Olsson H , Andersen M , Kadhim M , Helms G . MP3RAGE: simultaneous mapping of T_1_ and $B_{1}^{+}$ in human brain at 7T. Magn Reson Med. 2022;87:2637‐2649. doi:10.1002/mrm.29151 35037283

[28] Haast RAM , Lau JC , Ivanov D , Menon RS , Uludağ K , Khan AR . Effects of MP2RAGE $B_{1}^{+}$ sensitivity on inter‐site T_1_ reproducibility and hippocampal morphometry at 7T. Neuroimage. 2021;224:117373. doi:10.1016/j.neuroimage.2020.117373 32949709

[29] Forodighasemabadi A , Rasoanandrianina H , El Mendili MM , Guye M , Callot V . An optimized MP2RAGE sequence for studying both brain and cervical spinal cord in a single acquisition at 3T. Magn Reson Imaging. 2021;84:18‐26. doi:10.1016/j.mri.2021.08.011 34517015

[30] Kaipainen A , Jääskeläinen O , Liu Y , et al. Cerebrospinal fluid and MRI biomarkers in neurodegenerative diseases: a retrospective memory clinic‐based study. J Alzheimers Dis. 2020;75:751‐765. doi:10.3233/JAD-200175 32310181 PMC7369056

