# Supplementary material for: Improving B1+‐inhomogeneity tolerance by resolving non‐bijection in MP2RAGE R 1 mapping: A 2D look‐up table approach demonstrated at 3 T
Source: Magn Reson Med. 2024 Nov 11;93(4):1712–22. doi: 10.1002/mrm.30363 (PMC11782721; doi:10.1002/mrm.30363)
Supplement: Supplementary file 1 — Data S1 Supporting information. [file MRM-93-1712-s001.pdf]

Supporting Information for "A 3T investigation of B_1^+-inhomogeneity tolerance in MP2RAGE-based R_1-mapping by calculating second contrast that permits previously problematic sequence parameters"

September 6, 2024

Contents

List of Figures 1

List of Tables 5

1 Overview of acquisitions 8

2 How to change excitation flipangle to modify B_1^+ 9

3 R_1-nulling 10

4 The impact of curvature correction on R_1-maps 11

5 Robustness of CAT12 segmentation outputs 13

6 Robustness of R_1-values 16

7 Effect of B_1^+-correction. 26

8 A 7T example with 512 slices 34

List of Figures

S1 Modifying the excitation FA. Screenshot of Syngo scan card user interface (Siemens Healthineers, Erlangen, Germany) showing in orange highlights where SRFExcit was modified. 9

S2 R_1-nulling. Distribution of voxel-wise standard deviations across excitation pulse scalings over the mean $R_1$for the Standard (top) and LessBias (bottom) protocols. Increased density of the distribution is seen for $R_1 \approx 0.7\text{s}^{-1}$ (GM) and $R_1 \approx 1.15\text{s}^{-1}$ (WM) at lower standard deviations in LessBias compared to Standard, suggesting higher consistency across the R_1-estimations. Voids (white) in both distributions correspond with folds in the respective transfer curves ($R_1 < 0.26\text{s}^{-1}$ in Standard; $R_1 \approx 0.1\text{s}^{-1}$ (fold in the DSR transfer curve) or $R_1 \approx 0.4\text{s}^{-1}$ (fold in the UNI transfer curve) in LessBias), indicating R_1-nulling. 10

S3 The effect of curvature correction on the estimated R_1-surfaces and the standard deviation across B_1^+manipulations. By projecting out the local curvature of the R_1-values it is possible to remove curvature-induced variation in the R_1-mapping. As seen from the two bottom rows, the estimated curvatures are almost identical across protocols and B_1^+-manipulations. From the standard deviation of the curvature-uncorrected R_1-maps (two top rows) and curvature-corrected R_1-maps (two central rows), it is seen that these are entirely driven by the B_1^+-manipulations. 12

S4 GM R_1-assessment, Subject 1 Session 3, regarding multiple repetitions. Panel a) shows distributions of R_1-values on the central GM-surface for both Standard and LessBias, across three repetitions within the same scanning session. In panel b) the surfaces corresponding to the curves in panel a) are shown. In panel c) 2D histograms show the relationship between Standard and LessBias central, cortical GM R_1-values. The red lines indicate the perfect relationship between the data and the dashed, black lines indicate the best linear fit with an intercept of zero. 20

S5 GM R_1-assessment, Subject 1 Session 1-3, regarding multiple sessions. Panel a) shows distributions of R_1-values on the central GM-surface for both Standard and LessBias, across three scanning sessions. In panel b) the surfaces corresponding to the curves in panel a) are shown. In panel c) 2D histograms show the relationship between the Standard and the LessBias central, cortical GM R_1-values. The red lines indicate the perfect relationship between the data and the dashed, black lines indicate the best linear fit with an intercept of zero. . . 21

S6 GM R_1-assessment, Subject 2 Session 1-3, regarding multiple sessions. Panel a) shows the distribution of R_1-values on the central GM-surface for both Standard and LessBias across three scanning sessions. Panel b) shows the surfaces corresponding to the curves in panel a). Panel c) shows 2D histograms of the relationship between Standard and LessBias central GM cortical R_1-values. It is seen that both Standard and LessBias are quite consistent across sessions, although not quite at the level seen in subject1 Figure S5 where a less motion was present. See Table S5 for a whole brain comparison. 22

S7 GM R_1-assessment, Subject 3 Session 1, regarding multiple B_1^+-values. Panel a) shows distributions of R_1-values on the central GM-surface for both Standard and LessBias, across three B_1^+-manipulations. From the distributions and their median values (indicated with vertical lines) it is clear that LessBias method is quite consistent across B_1^+-values, but Standard fluctuates rapidly. Panel b) shows the surfaces corresponding to the curves in panel a). The Standard also visually shows a substantial fluctuation in R_1-values, whereas LessBias shows much less variation. Panel c) shows 2D histograms of the relationship between Standard and LessBias central GM cortical R_1-values. It is overall seen that LessBias presents with more constant distributions across the B_1^+-manipulations than Standard, and that Standard agrees most with LessBias when B_1^+: 100 %. 23
S8 GM R_1-assessment, Subject 2 Session 1, regarding number of slices. Panel a) shows distributions of R_1-values on the central GM-surface for both Standard and LessBias for the different number of slices. Panel b) shows the surfaces corresponding to the curves in panel a). Panel c) shows 2D histograms of the relationship between Standard and LessBias central GM cortical R_1-values. It is seen that in the examined interval, both protocols seem quite robust to changes in the number os slices. See Table S7 for a whole brain comparison. 24
S9 GM R_1-assessment, Subject 2 Session 3, regarding the fat navigator module. Panel a) shows distributions of R_1-values on the central GM-surface for both Standard and LessBias, with and without a fat navigator module present in the sequence. To be clear, "FatNavs" indicates that the acquisition was performed using the FatNavs-MP2RAGE sequence (Gallichan et al. MRM 75 (2016) 1030-1039), whereas "NoFatNavs" indicates that the MP2RAGE product version without fat navigators was used. The data in this analysis from the "FatNavs" scans, however, was the non-motion corrected data set. From the distributions (median values indicated with vertical lines) it is clear that the fat navigator pulse have no effect by itself. Panel b) shows the surfaces corresponding to the curves in panel a). In panel c), 2D histograms also show the lack of effect from the fat navigator module. The red lines indicate the perfect relationship between the data and the dashed, black lines indicate the best linear fit with an intercept of zero. An almost perfect correlation between the two MP2RAGE protocols is seen for both LessBias and Standard, indicating that the FatNavs pulse did not, by it self, influence the estimated R_1-values. 25
S10 Phantom B_1^+-maps. Instead of the original, ratio-based SA2RAGE method (three, top left images), we estimated our B_1^+-maps (three, top right images) through the I_{UNI}-values and performed the correct via a 3D-LUT procedure. The essential difference between the two procedures is shown in the three bottom left images. 27

S11 GM R_1-assessment, Subject 1 Session 2, regarding B_1^+-correction. Panel a) shows the distribution of R_1-values on the central GM-surface for both Standard and LessBias, As well as for a B_1^+-corrected version of Standard. From the distributions and their median values (indicated with vertical lines) it is clear that the R_1-values from Standard resemble those of LessBias, when the R_1-map from Standard is B_1^+-corrected. Panel b) shows the surfaces corresponding to the curves in panel a). A shift towards higher R_1-values at the cortex is seen, when the R_1-map from Standard is B_1^+-corrected. Panel c) shows 2D histograms for the three possible combinations of the maps in panel b). The red lines indicate the perfect relationship between the data and the dashed, black lines indicate the best linear fit with an intercept of zero. It is seen from the plots that while the maps from LessBias and Standard are fairly similar, the best correspondence is found between the R_1-maps from B_1^+-corrected Standard and LessBias. 28

S12 GM R_1-assessment, Subject 1 Session 3, regarding B_1^+-correction. Panel a) shows the distribution of R_1-values on the central GM-surface for both Standard and LessBias, As well as for a B_1^+-corrected version of Standard. From the distributions and their median values (indicated with vertical lines) it is clear that the R_1-values from Standard resemble those of LessBias, when the R_1-map from Standard is B_1^+-corrected. Panel b) shows the surfaces corresponding to the curves in panel a). A shift towards higher R_1-values at the cortex is seen, when the R_1-map from Standard is B_1^+-corrected. Panel c) shows 2D histograms for the three possible combinations of the maps in panel b). The red lines indicate the perfect relationship between the data and the dashed, black lines indicate the best linear fit with an intercept of zero. It is seen from the plots that while the maps from LessBias and Standard are fairly similar, the best correspondence is found between the R_1-maps from B_1^+-corrected Standard and LessBias. 29

S13 GM R_1-assessment, Subject 2 Session 1, regarding B_1^+-correction. Panel a) shows the distribution of R_1-values on the central GM-surface for both Standard and LessBias, As well as for a B_1^+-corrected version of Standard. From the distributions and their median values (indicated with vertical lines) it is clear that the R_1-values from Standard resemble those of LessBias, when the R_1-map from Standard is B_1^+-corrected. Panel b) shows the surfaces corresponding to the curves in panel a). A shift towards higher R_1-values at the cortex is seen, when the R_1-map from Standard is B_1^+-corrected. Panel c) shows 2D histograms for the three possible combinations of the maps in panel b). The red lines indicate the perfect relationship between the data and the dashed, black lines indicate the best linear fit with an intercept of zero. It is seen from the plots that while the maps from LessBias and Standard are fairly similar, the best correspondence is found between the R_1-maps from B_1^+-corrected Standard and LessBias. 30

S14 GM R_1-assessment, Subject 2 Session 2, regarding B_1^+-correction. Panel a) shows the distribution of R_1-values on the central GM-surface for both Standard and LessBias, As well as for a B_1^+-corrected version of Standard. From the distributions and their median values (indicated with vertical lines) it is clear that the R_1-values from Standard resemble those of LessBias, when the R_1-map from Standard is B_1^+-corrected. Panel b) shows the surfaces corresponding to the curves in panel a). A shift towards higher R_1-values at the cortex is seen, when the R_1-map from Standard is B_1^+-corrected. Panel c) shows 2D histograms for the three possible combinations of the maps in panel b). The red lines indicate the perfect relationship between the data and the dashed, black lines indicate the best linear fit with an intercept of zero. It is seen from the plots that while the maps from LessBias and Standard are fairly similar, the best correspondence is found between the R_1-maps from B_1^+-corrected Standard and LessBias. 31

S15 GM R_1-assessment, Subject 3 Session 1, regarding B_1^+-correction. Panel a) shows the distribution of R_1-values on the central GM-surface for both Standard and LessBias, As well as for a B_1^+-corrected version of Standard. From the distributions and their median values (indicated with vertical lines) it is clear that the R_1-values from Standard resemble those of LessBias, when the R_1-map from Standard is B_1^+-corrected. Panel b) shows the surfaces corresponding to the curves in panel a). A shift towards higher R_1-values at the cortex is seen, when the R_1-map from Standard is B_1^+-corrected. Panel c) shows 2D histograms for the three possible combinations of the maps in panel b). The red lines indicate the perfect relationship between the data and the dashed, black lines indicate the best linear fit with an intercept of zero. It is seen from the plots that while the maps from LessBias and Standard are fairly similar, the best correspondence is found between the R_1-maps from B_1^+-corrected Standard and LessBias. 32

S16 GM R_1-assessment, Subject 2 Session 1, regarding B_1^+-correction. Panel a) shows distributions of R_1-values on the central GM-surface for both protocols, with and without B_1^+-correction. From the distributions and their median values (indicated with vertical lines) it is clear that the R_1-values from Standard resemble those of LessBias, when the R_1-map from Standard is B_1^+-corrected. However, as could be expected from Figure 5, the effect of B_1^+-correction on LessBias is minimal. Panel b) shows the surfaces corresponding to the curves in panel a). A shift towards higher R_1-values at the cortex is seen, when the R_1-map from Standard is B_1^+-corrected. In panel c), 2D histograms show the effect of B_1^+-correction for the two different protocols. The red lines indicate the perfect relationship between the data and the dashed, black lines indicate the best linear fit with an intercept of zero. It is seen that LessBias and LessBias with B_1^+-correction have a stronger correlation than Standard and Standard with B_1^+-correction. Panel d) shows the B_1^+-map used for the correction. 33

S17 Transfer functions for two protocols **a**: Standard* and **b**: LessBias* modified to acquire 512 slices at 7T. 34

List of Tables

S1 Supporting Figures overview with respect to investigated features. A • indicates a feature that has been investigated. 8

S2 Segmentation robustness within and across sessions. Top (Subject 1 Session 3) shows the variation of estimated volumes across repeated acquisitions of the two protocols. It is seen that LessBias shows a 2-3 fold larger standard deviation in estimated volumes, when compared to Standard, but in both cases the standard deviation across repetitions are for GM approximately 0.25 % of the corresponding volumes. Center (Subject 1) and bottom (Subject 2) show that Standard yields a 2.68 and 1.7 times higher standard deviation in the GM volume estimation than LessBias, respectively, when compared different sessions of the same subject. 13
S3 Segmentation robustness across subjects. GM, WM, CSF and TIV volumes and GM average thickness for different protocols, subjects and B_1^+-manipulations from four sessions in three subjects. It is consistently seen that while Standard shows substantial variation in estimated volumes across B_1^+-modifications, LessBias is practically immune to the B_1^+-alterations. The standard deviation seen in the estimated volumes for LessBias is at the level of that seen in the top of Table S4 where each protocol was repeated three times without B_1^+-modifications. For Standard on the other hand the standard deviation across B_1^+-alterations is up to 22 times larger than what is seen for LessBias. 14
S4 Segmentation robustness across sequence parameters. Top (Subject 2 Session 1), it is seen that the estimated volumes for the two protocols change a lot when Standard is acquired with various number of slices, while this is not the case for LessBias. The standard deviation across slice modulations for Standard is 2-16 times greater than for LessBias, where the standard deviation is comparable to when no parameters are modified. In GM, the standard deviation of estimated GM volumes for Standard is almost 2% of the estimated volume. At the bottom (Subject 2 Session 3), the effect of B_1^+correction and presence of fat navigators (FatNavs) are shown. A • in the FatNavs column indicates that the acquisition was performed using the FatNavs-MP2RAGE sequence (Gallichan et al. MRM 75 (2016) 1030-1039), whereas a ◦ indicates that the product version without fat navigators was used. Both protocols were reconstructed on the scanner i.e. without performing the motion correction. For LessBias, the estimated volumes are practically unaffected by the fat navigators, while the standard deviation for Standard is 8 times larger than for LessBias. In GM, the standard deviation of estimated GM volumes for Standard is around 1% of the estimated volume. B_1^+-correction of Standard reduce the standard deviation between the protocols by a factor of 2. 15
S5 R_1-values (mean and standard deviation) within and across sessions. 17
S6 R_1-values (mean and standard deviation) across subjects. In all four sessions Standard and LessBias were subject to B_1^+manipulations through changes of the FA (60, 100, and 140 %), see Sec. 2. We see a substantially smaller R_1standard deviation for LessBias in GM and WM than for Standard. Improvements range from a factor of 3.3 to 21.5. For CSF, LessBias ranges from 2 to 1.04 times higher than that of Standard. 18

S7 R_1-values (mean and standard deviation) across sequence parameters. In the top (Subject 2 Session 1), the number of slices were changed. In the bottom (Subject 2 Session 3) a • in the FatNavs column indicates that the acquisition was performed using the FatNavs-MP2RAGE sequence (Gallichan et al. MRM 75 (2016) 1030-1039), whereas a ◦ indicates that the product version without fat navigators was used. Both sequences were reconstructed on the scanner i.e. without performing the motion correction. All scans were obtained with the same 100 % FA setting, and we see that the standard deviations for all tissue types are below 0.007 s^{-1} for both Standard and LessBias. 19

1 Overview of acquisitions

In order to explore the robustness of the 2D-LUT and the LessBias protocols a total of seven scanning sessions were performed on three subjects. In addition to the protocol presented in the paper, the following four variations were tested: 1) The possible impact of the curvature correction performed when sampling R_1-values at the central GM surface. 2) Three repeated acquisitions each of the two protocols (Standard and LessBias) without modifying the B_1^+ at the stage of acquisition. This session (Subject 1, Session 3) was used to evaluate the relation between image noise and B_1^+-introduced bias. 3) Three different number of slices 160, 192 and 240 were used with each of the two protocols (Standard and LessBias). 4) Potential magnetisation transfer effect of fat navigators (FatNavs) were evaluated in a third session where both the Standard and LessBias protocol were acquired with and without fat navigators. An overview of the different sessions is presented in Table S1 below.

Table S1: Supporting Figures overview with respect to investigated features. A • indicates a feature that has been investigated.

	Subject	Session	B_1^+-manip.	Repeat	#Slices	FatNavs	B_1^+-corr.	Curv.-corr
Figure S3	1	1	○	○	○	○	○	•
Figure S4	1	3	○	•	○	○	○	○
Figure S5	1	1,2,3	○	○	○	○	○	○
Figure S6	2	1,2,3	○	○	○	○	○	○
Figure S7	3	1	•	○	○	○	○	○
Figure S8	2	1	○	○	•	○	○	○
Figure S9	2	3	○	○	○	•	○	○
Figure S11	1	2	○	○	○	○	•	○
Figure S12	1	3	○	○	○	○	•	○
Figure S13	2	1	○	○	○	○	•	○
Figure S14	2	2	○	○	○	○	•	○
Figure S15	3	1	○	○	○	○	•	○
Figure S16	2	1	○	○	○	○	• •	○

2 How to change excitation flipangle to modify B_1^+

If the scanner interface had allowed for non-integer flipangles (FAs) the ±40% modifications of B_1^+ could have been implemented by just entering the corresponding FAs. As this is not possible, we modified the SRFExcit pulse amplitude as shown in Figure S1.

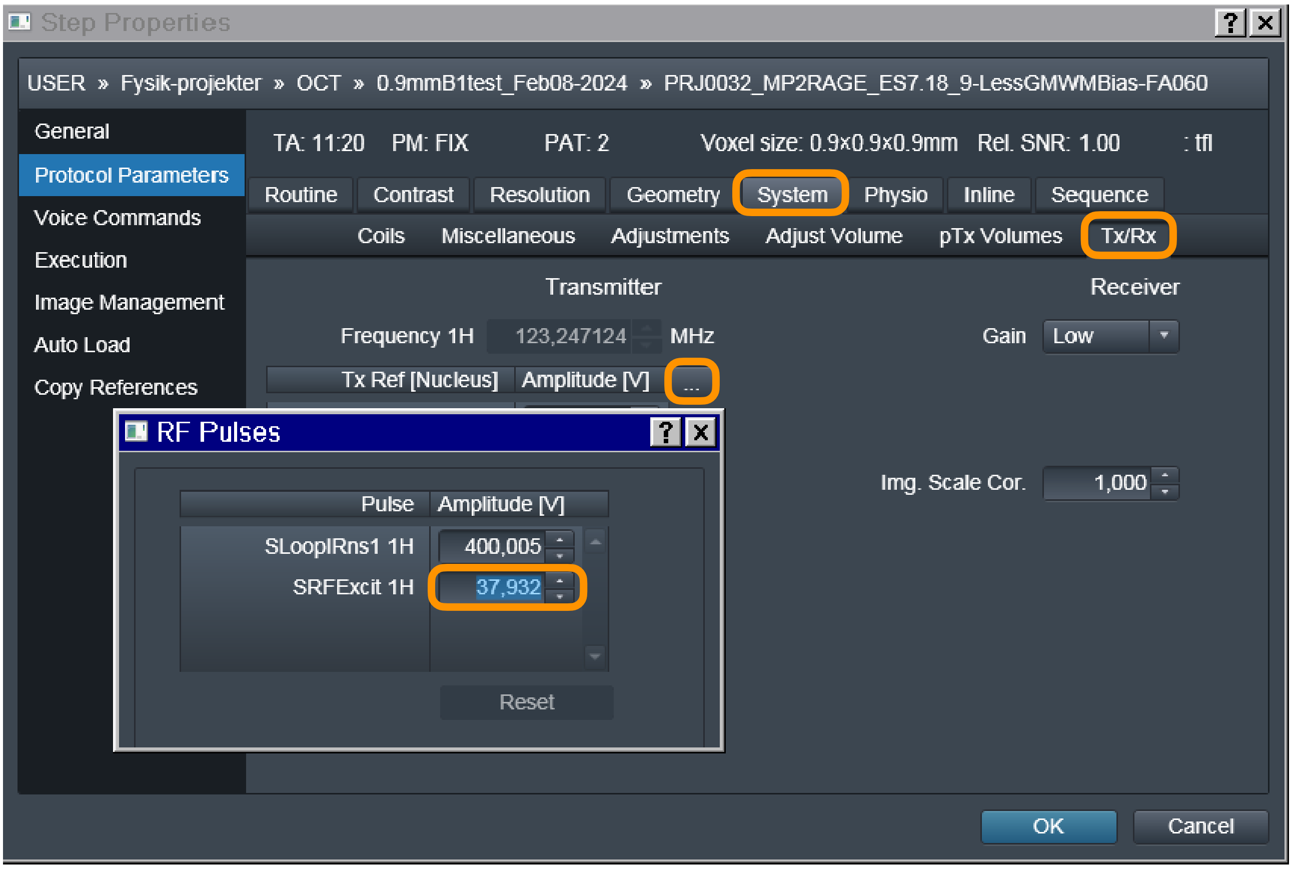

Figure S1: Modifying the excitation FA. Screenshot of Syngo scan card user interface (Siemens Healthineers, Erlangen, Germany) showing in orange highlights where SRFExcit was modified.

3 R_1-nulling

We observed a phenomenon we dubbed R_1-nulling, that is present in both 1D- and 2D-LUT R_1-mapping procedures. Certain R_1-values will not be assigned to voxels with certain characteristics. This can be seen in Figure S2, where white gaps appear in the distribution of R_1-values. Firstly, we noticed that this occurs for R_1-values close to the fold of the transfer curve (i.e. when I_{UNI}= -0.5). We speculate that this is the result of noise in the first and second inversion images. When computing the UNI values from these images, noise in either direction in the inversion images will necessarily always push the UNI-value closer to 0, whereas the UNI-values for voxels farther away from the boundaries of UNI can go either way as a result of noise. As a result, we expect that the SNR will have a substantial influence on the severity of this R1-nulling phenomenon. Both protocols avoid imposing this problem on the R1-maps by making sure I_{UNI}= -0.5 for R_1-values that are removed from tissue if interest (for Standard, at $R_1 \approx 0.12\text{s}^{-1}$, below CSF; for LessBias at $R_1 \approx 0.4\text{s}^{-1}$, between the expected R_1-values for CSF and GM). Note that for certain future purposes, like selective contrast, it may be difficult to avoid R_1-nulling in critical R_1-values, as a result of the location of the fold in the transfer curve or limitations of the SNR. More sophisticated algorithms may prove equipped to handle such data, and are under investigation.

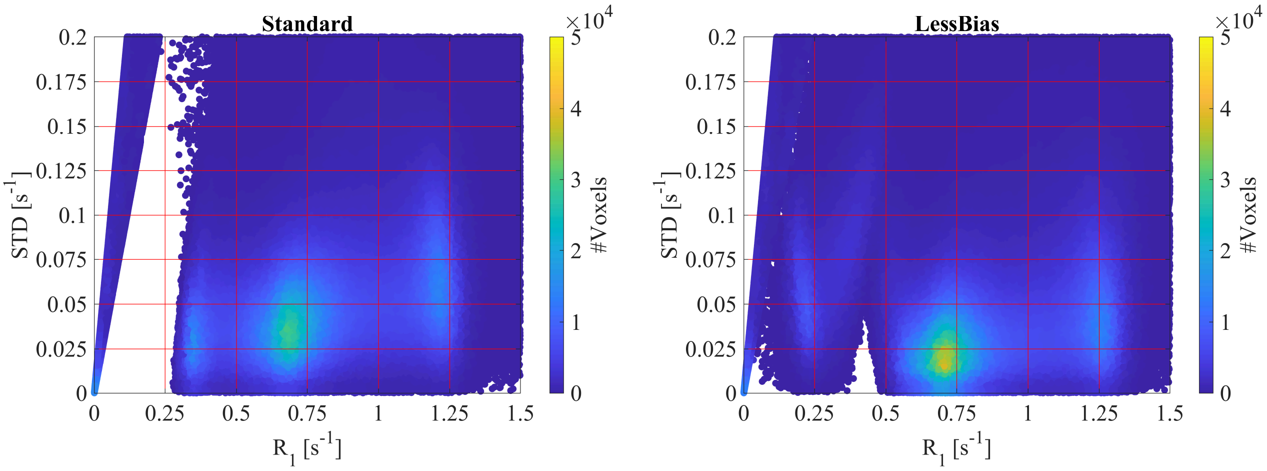

Figure S2: R_1-nulling. Distribution of voxel-wise standard deviations across excitation pulse scalings over the mean $R_1$for the Standard (top) and LessBias (bottom) protocols. Increased density of the distribution is seen for $R_1 \approx 0.7\text{s}^{-1}$ (GM) and $R_1 \approx 1.15\text{s}^{-1}$ (WM) at lower standard deviations in LessBias compared to Standard, suggesting higher consistency across the R_1-estimations. Voids (white) in both distributions correspond with folds in the respective transfer curves ($R_1 < 0.26\text{s}^{-1}$ in Standard; $R_1 \approx 0.1\text{s}^{-1}$ (fold in the DSR transfer curve) or $R_1 \approx 0.4\text{s}^{-1}$ (fold in the UNI transfer curve) in LessBias), indicating R_1-nulling.

4 The impact of curvature correction on R_1-maps

As the myelination of the cortex is influenced trivially by the local curvature, it makes sense to perform a correction for the local curvature, when sampling R_1-maps on the cortical surface. To illustrate that the effects of our B_1^+-manipulations are not affected by this, we show in Figure S3 both the curvature-corrected and uncorrected R_1-surfaces together with the estimated curvature. From the standard deviation surfaces it is seen that the variation in the estimated curvature across B_1^+-manipulation does not affect the R_1-maps.

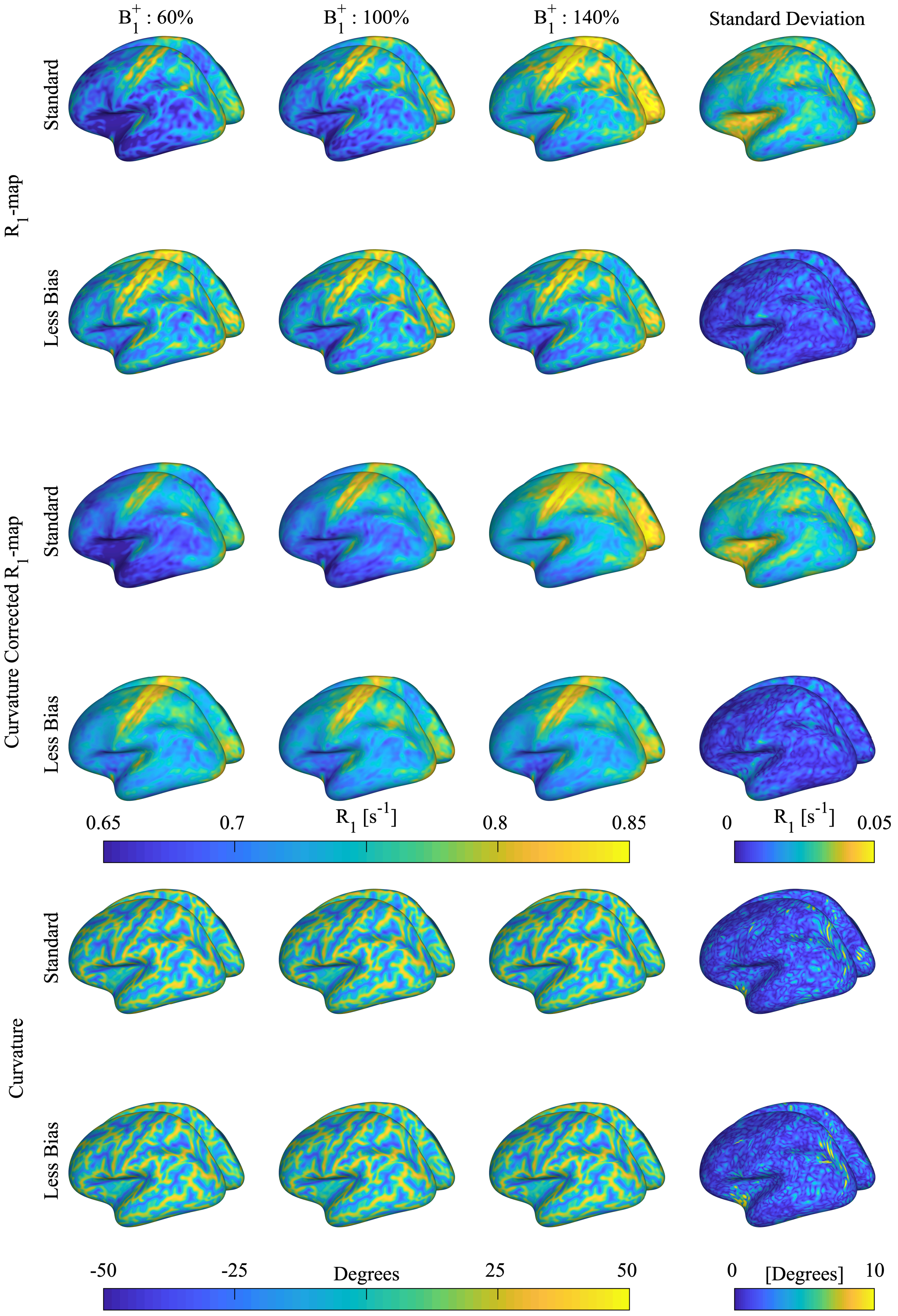

Figure S3: The effect of curvature correction on the estimated R_1-surfaces and the standard deviation across B_1^+manipulations. By projecting out the local curvature of the R_1-values it is possible to remove curvature-induced variation in the R_1-mapping. As seen from the two bottom rows, the estimated curvatures are almost identical across protocols and B_1^+-manipulations. From the standard deviation of the curvature-uncorrected R_1-maps (two top rows) and curvature-corrected R_1-maps (two central rows), it is seen that these are entirely driven by the B_1^+-manipulations.

5 Robustness of CAT12 segmentation outputs

The volumes and thickness estimates outputted from the CAT12 segmentation was assessed towards robustness within and between sessions (Table S2), between subjects and B_1^+-manipulations (Table S3); and across sequence parameters)Table S4). From these tables it is clear that LessBias offers a substantial improvement in the consistency of the outputted tissue volumes, when B_1^+or sequence parameters are altered.

Table S2: Segmentation robustness within and across sessions. Top (Subject 1 Session 3) shows the variation of estimated volumes across repeated acquisitions of the two protocols. It is seen that LessBias shows a 2-3 fold larger standard deviation in estimated volumes, when compared to Standard, but in both cases the standard deviation across repetitions are for GM approximately 0.25 % of the corresponding volumes. Center (Subject 1) and bottom (Subject 2) show that Standard yields a 2.68 and 1.7 times higher standard deviation in the GM volume estimation than LessBias, respectively, when compared different sessions of the same subject.

**Subject 1 Session 3**	Rep.	CSF [mL]	GM [mL]	WM [mL]	TIV [mL]	GM-thickness [mm]
Standard	1	310.5	816.9	524.9	1652.3	2.34
	2	311.2	816.6	524.4	1652.2	2.31
	3	311.9	818.0	524.8	1654.7	2.31
LessBias	1	259.1	852.7	523.4	1635.1	2.40
	2	255.1	855.7	524.3	1635.1	2.40
	3	258.5	851.2	523.9	1633.6	2.44
**Std(Standard)**	-	**0.7**	**0.7**	**0.3**	**1.4**	**0.01**
**Std(LessBias)**	-	**2.1**	**2.3**	**0.5**	**0.9**	**0.03**

**Subject 1**	Session	CSF [mL]	GM [mL]	WM [mL]	TIV [mL]	GM-thickness [mm]
Standard	1	302.7	828.4	524.8	1655.9	2.31
	2	304.4	823.2	525.6	1653.2	2.34
	3	311.2	816.6	524.4	1652.2	2.31
LessBias	1	251.3	859.5	523.0	1633.8	2.43
	2	251.1	859.4	524.8	1635.3	2.41
	3	255.1	855.7	524.3	1635.1	2.40
**Std(Standard)**	-	**4.5**	**5.9**	**0.6**	**1.9**	**0.01**
**Std(LessBias)**	-	**2.3**	**2.2**	**0.9**	**0.8**	**0.02**

**Subject 2**	Session	CSF [mL]	GM [mL]	WM [mL]	TIV [mL]	GM-thickness [mm]
Standard	1	283.0	828.9	644.1	1756.1	2.41
	2	261.9	848.9	647.4	1758.3	2.38
	3	259.6	852.5	649.1	1761.1	2.46
LessBias	1	212.3	880.9	645.3	1738.5	2.36
	2	205.1	892.7	646.7	1744.5	2.35
	3	203.8	878.5	642.6	1724.9	2.58
**Std(Standard)**	-	**12.9**	**12.7**	**2.5**	**2.5**	**0.04**
**Std(LessBias)**	-	**4.6**	**7.6**	**2.1**	**10.1**	**0.13**

Table S3: Segmentation robustness across subjects. GM, WM, CSF and TIV volumes and GM average thickness for different protocols, subjects and B_1^+-manipulations from four sessions in three subjects. It is consistently seen that while Standard shows substantial variation in estimated volumes across B_1^+-modifications, LessBias is practically immune to the B_1^+-alterations. The standard deviation seen in the estimated volumes for LessBias is at the level of that seen in the top of Table S4 where each protocol was repeated three times without B_1^+-modifications. For Standard on the other hand the standard deviation across B_1^+-alterations is up to 22 times larger than what is seen for LessBias.

**Subject 1 Session 1**	B_1^+ [%]	CSF [mL]	GM [mL]	WM [mL]	TIV [mL]	GM-thickness [mm]
Standard	60	342.8	778.0	521.1	1642.0	2.16
	100	302.7	828.4	524.8	1655.9	2.31
	140	289.8	847.2	524.0	1661.0	2.38
LessBias	60	233.5	868.1	521.9	1623.5	2.56
	100	251.3	859.5	523.0	1633.8	2.43
	140	257.6	857.7	522.7	1638.0	2.40
**Std(Standard)**	-	**27.7**	**35.8**	**1.9**	**9.8**	**0.11**
**Std(LessBias)**	-	**12.5**	**5.5**	**0.6**	**7.4**	**0.08**

**Subject 1 Session 2**	B_1^+ [%]	CSF [mL]	GM [mL]	WM [mL]	TIV [mL]	GM-thickness [mm]
Standard	60	342.2	779.9	522.9	1645.0	2.20
	100	304.4	823.2	525.6	1653.2	2.34
	140	292.8	843.2	525.5	1661.5	2.38
LessBias	60	245.3	861.5	524.1	1630.9	2.45
	100	251.1	859.4	524.8	1635.3	2.41
	140	260.5	858.6	522.2	1641.3	2.31
**Std(Standard)**	-	**25.9**	**32.4**	**1.5**	**8.2**	**0.09**
**Std(LessBias)**	-	**7.7**	**1.5**	**1.3**	**5.2**	**0.07**

**Subject 2 Session 2**	B_1^+ [%]	CSF [mL]	GM [mL]	WM [mL]	TIV [mL]	GM-thickness [mm]
Standard	60	304.0	796.2	646.8	1747.0	2.28
	100	261.9	848.9	647.4	1758.3	2.38
	140	245.0	866.5	648.5	1760.0	2.43
LessBias	60	192.7	894.0	648.0	1734.7	2.48
	100	205.1	892.7	646.7	1744.5	2.35
	140	204.5	895.8	643.1	1743.4	2.37
**Std(Standard)**	-	**30.4**	**36.6**	**0.9**	**7.1**	**0.08**
**Std(LessBias)**	-	**7.0**	**1.6**	**2.5**	**5.4**	**0.07**

**Subject 3 Session 1**	B_1^+ [%]	CSF [mL]	GM [mL]	WM [mL]	TIV [mL]	GM-thickness [mm]
Standard	60	287.5	803.7	563.6	1654.8	2.32
	100	267.6	835.6	561.6	1664.7	2.44
	140	253.9	857.3	560.5	1671.7	2.62
LessBias	60	192.2	886.4	565.3	1643.9	2.59
	100	207.3	885.7	558.8	1651.8	2.56
	140	208.3	887.8	558.0	1654.1	2.52
**Std(Standard)**	-	**16.9**	**27.0**	**1.6**	**8.5**	**0.15**
**Std(LessBias)**	-	**9.0**	**1.1**	**4.0**	**5.3**	**0.03**

Table S4: Segmentation robustness across sequence parameters. Top (Subject 2 Session 1), it is seen that the estimated volumes for the two protocols change a lot when Standard is acquired with various number of slices, while this is not the case for LessBias. The standard deviation across slice modulations for Standard is 2-16 times greater than for LessBias, where the standard deviation is comparable to when no parameters are modified. In GM, the standard deviation of estimated GM volumes for Standard is almost 2% of the estimated volume. At the bottom (Subject 2 Session 3), the effect of B_1^+correction and presence of fat navigators (FatNavs) are shown. A • in the FatNavs column indicates that the acquisition was performed using the FatNavs-MP2RAGE sequence (Gallichan et al. MRM 75 (2016) 1030-1039), whereas a ○ indicates that the product version without fat navigators was used. Both protocols were reconstructed on the scanner i.e. without performing the motion correction. For LessBias, the estimated volumes are practically unaffected by the fat navigators, while the standard deviation for Standard is 8 times larger than for LessBias. In GM, the standard deviation of estimated GM volumes for Standard is around 1% of the estimated volume. B_1^+-correction of Standard reduce the standard deviation between the protocols by a factor of 2.

**Subject 2 Session 1**	#Slices	CSF [mL]	GM [mL]	WM [mL]	TIV [mL]	GM-thickness [mm]
Standard	160	291.6	814.1	642.1	1747.9	2.40
	192	283.0	828.9	644.1	1756.1	2.41
	240	269.0	844.6	645.9	1759.5	2.45
LessBias	160	213.9	881.2	643.6	1738.7	2.39
	192	212.3	880.9	645.3	1738.5	2.36
	240	215.9	882.4	646.1	1744.4	2.37
**Std(Standard)**	-	**11.4**	**15.2**	**1.9**	**5.9**	**0.03**
**Std(LessBias)**	-	**1.8**	**0.8**	**1.3**	**3.3**	**0.01**

**Subject 2 Session 3**	FatNavs	CSF [mL]	GM [mL]	WM [mL]	TIV [mL]	GM-Thickness [mm]
Standard	•	259.6	852.5	649.1	1761.1	2.5
	○	265.7	840.8	648.4	1754.8	2.5
LessBias	•	203.8	878.5	642.6	1724.9	2.6
	○	203.0	877.0	642.6	1722.7	2.6
**Std(Standard)**	-	**4.3**	**8.3**	**0.5**	**4.4**	**0.03**
**Std(LessBias)**	-	**0.6**	**1.0**	**0.0**	**1.6**	**0.01**

6 Robustness of R_1-values

The resulting R_1-values (mean and standard deviation), from images segmented with CAT12, were assessed towards robustness within and across sessions (Table S5); across subjects and B_1^+-manipulations (Table S6); and sequence parameters, (Table S7). For each tissue type (GM, WM and CSF), a mask was created from the intersections between Standard and LessBias of the segmented volumes from the unmanipulated (B1+: 100%) Standard and LessBias images. In Figure S4, we show robustness of R_1-surfaces, togeher with 1D and 2D histograms for repeated acquisitions within the same session, and in Figure S5 and Figure S6 for repetition across multiple sessions. The surfaces in Figure S7 are the same as those found in Figure 4 of the main paper. From the 1D and 2D histograms, we can further appreciate the effects of the B_1^+-manipulations described in the paper. From the 1D histogram, it is confirmed that the LessBias protocol is indeed very robust to the B_1^+-manipulations, whereas the Standard protocol is very affected. In Figure S8, we confirm that the R1 estimates are robust to changes in the number of slices in the interval from 160 to 240. Finally in Figure S9, we confirm that our use of FatNavs are unlikely to have introduced any bias to our R_1-mapping.

Table S5: R_1-values (mean and standard deviation) within and across sessions.

**Subject 1 Session 3**	Rep.	CSF [s^{-1}]	GM [s^{-1}]	WM [s^{-1}]
Standard	1	0.401 ± 0.170	0.712 ± 0.079	1.186 ± 0.077
	2	0.400 ± 0.173	0.712 ± 0.077	1.188 ± 0.079
	3	0.402 ± 0.176	0.712 ± 0.079	1.184 ± 0.078
LessBias	1	0.328 ± 0.265	0.732 ± 0.084	1.228 ± 0.089
	2	0.333 ± 0.272	0.734 ± 0.088	1.226 ± 0.090
	3	0.335 ± 0.274	0.734 ± 0.087	1.225 ± 0.089
**Standard: Mean ± Std**	-	**0.401 ± 0.001**	**0.712 ± 0.000**	**1.186 ± 0.002**
**LessBias: Mean ± Std**	-	**0.332 ± 0.004**	**0.733 ± 0.001**	**1.226 ± 0.002**

**Subject 1**	Session	CSF [s^{-1}]	GM [s^{-1}]	WM [s^{-1}]
Standard	1	0.398 ± 0.170	0.710 ± 0.077	1.186 ± 0.078
	2	0.400 ± 0.180	0.710 ± 0.077	1.188 ± 0.078
	3	0.401 ± 0.178	0.711 ± 0.077	1.188 ± 0.079
LessBias	1	0.324 ± 0.263	0.731 ± 0.084	1.228 ± 0.087
	2	0.329 ± 0.278	0.729 ± 0.084	1.225 ± 0.086
	3	0.328 ± 0.273	0.732 ± 0.083	1.228 ± 0.088
**Standard: Mean ± Std**	-	**0.400 ± 0.002**	**0.710 ± 0.001**	**1.187 ± 0.001**
**LessBias: Mean ± Std**	-	**0.327 ± 0.003**	**0.731 ± 0.002**	**1.227 ± 0.002**

**Subject 2**	Session	CSF [s^{-1}]	GM [s^{-1}]	WM [s^{-1}]
Standard	1	0.439 ± 0.209	0.713 ± 0.086	1.219 ± 0.081
	2	0.453 ± 0.239	0.705 ± 0.084	1.207 ± 0.077
	3	0.423 ± 0.179	0.711 ± 0.080	1.208 ± 0.072
LessBias	1	0.380 ± 0.327	0.733 ± 0.089	1.262 ± 0.093
	2	0.399 ± 0.347	0.725 ± 0.090	1.251 ± 0.087
	3	0.353 ± 0.253	0.727 ± 0.082	1.250 ± 0.080
**Standard: Mean ± Std**	-	**0.438 ± 0.015**	**0.710 ± 0.004**	**1.211 ± 0.006**
**LessBias: Mean ± Std**	-	**0.378 ± 0.023**	**0.728 ± 0.004**	**1.254 ± 0.007**

Table S6: R_1-values (mean and standard deviation) across subjects. In all four sessions Standard and LessBias were subject to B_1^+manipulations through changes of the FA (60, 100, and 140 %), see Sec. 2. We see a substantially smaller R_1standard deviation for LessBias in GM and WM than for Standard. Improvements range from a factor of 3.3 to 21.5. For CSF, LessBias ranges from 2 to 1.04 times higher than that of Standard.

**Subject 1 Session 1**	B_1^+ [%]	CSF [s^{-1}]	GM [s^{-1}]	WM [s^{-1}]
Standard	60	0.432 ± 0.145	0.681 ± 0.078	1.135 ± 0.085
	100	0.391 ± 0.164	0.709 ± 0.077	1.186 ± 0.078
	140	0.404 ± 0.179	0.750 ± 0.084	1.254 ± 0.079
LessBias	60	0.313 ± 0.328	0.742 ± 0.088	1.219 ± 0.098
	100	0.318 ± 0.256	0.731 ± 0.087	1.227 ± 0.090
	140	0.353 ± 0.250	0.732 ± 0.086	1.251 ± 0.090
**Standard: Mean ± Std**	-	**0.409 ± 0.021**	**0.713 ± 0.035**	**1.192 ± 0.060**
**LessBias: Mean ± Std**	-	**0.328 ± 0.022**	**0.735 ± 0.006**	**1.232 ± 0.017**

**Subject 1 Session 2**	B_1^+ [%]	CSF [s^{-1}]	GM [s^{-1}]	WM [s^{-1}]
Standard	60	0.427 ± 0.157	0.686 ± 0.078	1.136 ± 0.082
	100	0.397 ± 0.176	0.710 ± 0.077	1.187 ± 0.079
	140	0.420 ± 0.191	0.768 ± 0.087	1.285 ± 0.084
LessBias	60	0.311 ± 0.314	0.737 ± 0.088	1.216 ± 0.095
	100	0.327 ± 0.272	0.730 ± 0.088	1.223 ± 0.088
	140	0.372 ± 0.265	0.736 ± 0.088	1.259 ± 0.092
**Standard: Mean ± Std**	-	**0.415 ± 0.016**	**0.721 ± 0.042**	**1.203 ± 0.076**
**LessBias: Mean ± Std**	-	**0.337 ± 0.032**	**0.734 ± 0.004**	**1.233 ± 0.023**

**Subject 2 Session 2**	B_1^+ [%]	CSF [s^{-1}]	GM [s^{-1}]	WM [s^{-1}]
Standard	60	0.468 ± 0.192	0.678 ± 0.088	1.150 ± 0.087
	100	0.435 ± 0.223	0.706 ± 0.085	1.207 ± 0.077
	140	0.453 ± 0.228	0.745 ± 0.093	1.269 ± 0.083
LessBias	60	0.376 ± 0.387	0.734 ± 0.096	1.238 ± 0.101
	100	0.380 ± 0.328	0.727 ± 0.096	1.249 ± 0.090
	140	0.415 ± 0.315	0.731 ± 0.094	1.272 ± 0.095
**Standard: Mean ± Std**	-	**0.452 ± 0.017**	**0.709 ± 0.034**	**1.209 ± 0.059**
**LessBias: Mean ± Std**	-	**0.390 ± 0.021**	**0.731 ± 0.004**	**1.253 ± 0.017**

**Subject 3 Session 1**	B_1^+ [%]	CSF [s^{-1}]	GM [s^{-1}]	WM [s^{-1}]
Standard	60	0.446 ± 0.162	0.669 ± 0.075	1.100 ± 0.076
	100	0.416 ± 0.181	0.695 ± 0.075	1.147 ± 0.073
	140	0.440 ± 0.194	0.753 ± 0.085	1.243 ± 0.081
LessBias	60	0.341 ± 0.344	0.719 ± 0.080	1.173 ± 0.087
	100	0.344 ± 0.290	0.715 ± 0.079	1.182 ± 0.082
	140	0.384 ± 0.272	0.715 ± 0.077	1.209 ± 0.084
**Standard: Mean ± Std**	-	**0.434 ± 0.016**	**0.706 ± 0.043**	**1.163 ± 0.073**
**LessBias: Mean ± Std**	-	**0.356 ± 0.024**	**0.716 ± 0.002**	**1.188 ± 0.019**

Table S7: R_1-values (mean and standard deviation) across sequence parameters. In the top (Subject 2 Session 1), the number of slices were changed. In the bottom (Subject 2 Session 3) a • in the FatNavs column indicates that the acquisition was performed using the FatNavs-MP2RAGE sequence (Gallichan et al. MRM 75 (2016) 1030-1039), whereas a ○ indicates that the product version without fat navigators was used. Both sequences were reconstructed on the scanner i.e. without performing the motion correction. All scans were obtained with the same 100 % FA setting, and we see that the standard deviations for all tissue types are below 0.007 s^{-1} for both Standard and LessBias.

**Subject 2 Session 1**	#Slices	CSF [s^{-1}]	GM [s^{-1}]	WM [s^{-1}]
Standard	160	0.457 ± 0.206	0.714 ± 0.084	1.216 ± 0.082
	192	0.439 ± 0.209	0.713 ± 0.086	1.219 ± 0.081
	240	0.429 ± 0.232	0.712 ± 0.088	1.219 ± 0.081
LessBias	160	0.381 ± 0.317	0.738 ± 0.088	1.267 ± 0.092
	192	0.380 ± 0.327	0.733 ± 0.089	1.262 ± 0.093
	240	0.394 ± 0.351	0.730 ± 0.095	1.263 ± 0.092
**Standard: Mean ± Std**	-	**0.442 ± 0.014**	**0.713 ± 0.001**	**1.218 ± 0.002**
**LessBias: Mean ± Std**	-	**0.385 ± 0.008**	**0.734 ± 0.004**	**1.264 ± 0.003**

**Subject 2 Session 3**	FatNavs	CSF [s^{-1}]	GM [s^{-1}]	WM [s^{-1}]
Standard	•	0.423 ± 0.179	0.711 ± 0.080	1.208 ± 0.072
	○	0.425 ± 0.178	0.703 ± 0.082	1.199 ± 0.074
LessBias	•	0.353 ± 0.253	0.727 ± 0.082	1.250 ± 0.080
	○	0.359 ± 0.251	0.725 ± 0.085	1.243 ± 0.083
**Standard: Mean ± Std**	-	**0.424 ± 0.001**	**0.707 ± 0.006**	**1.204 ± 0.007**
**LessBias: Mean ± Std**	-	**0.356 ± 0.004**	**0.726 ± 0.002**	**1.246 ± 0.006**

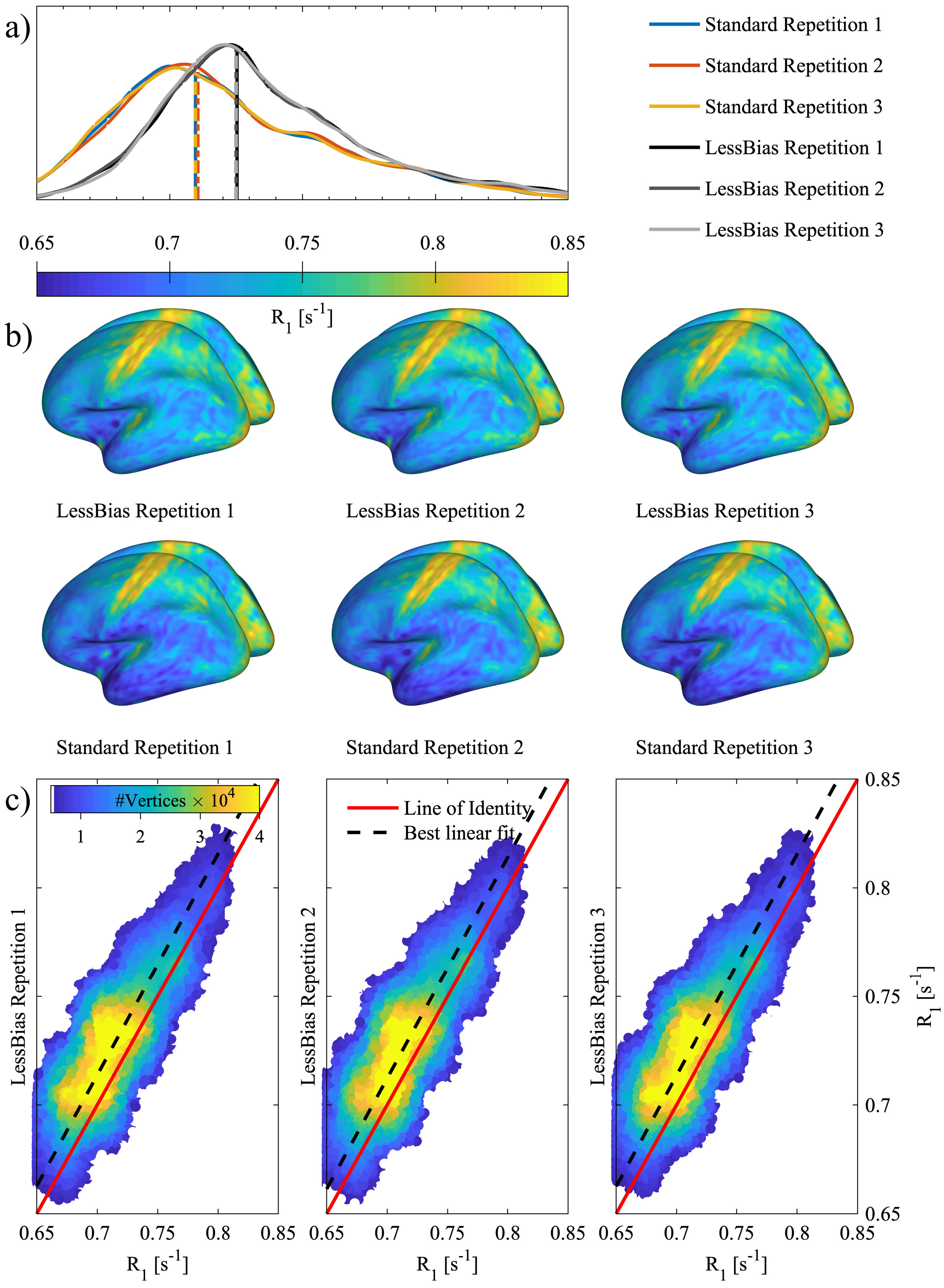

Figure S4: GM R_1-assessment, Subject 1 Session 3, regarding multiple repetitions. Panel a) shows distributions of R_1-values on the central GM-surface for both Standard and LessBias, across three repetitions within the same scanning session. In panel b) the surfaces corresponding to the curves in panel a) are shown. In panel c) 2D histograms show the relationship between Standard and LessBias central, cortical GM R_1-values. The red lines indicate the perfect relationship between the data and the dashed, black lines indicate the best linear fit with an intercept of zero.

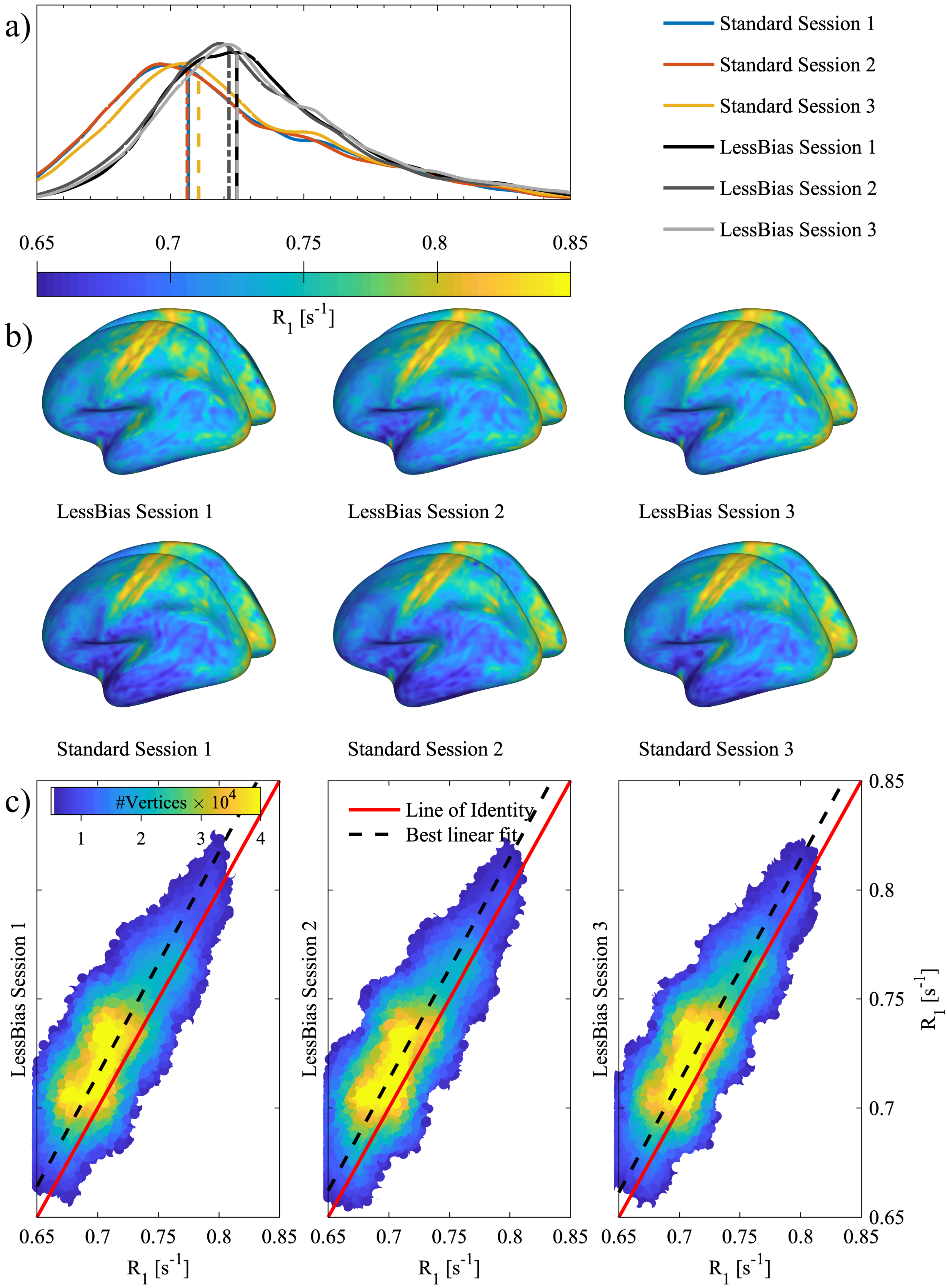

Figure S5: GM R_1-assessment, Subject 1 Session 1-3, regarding multiple sessions. Panel a) shows distributions of R_1-values on the central GM-surface for both Standard and LessBias, across three scanning sessions. In panel b) the surfaces corresponding to the curves in panel a) are shown. In panel c) 2D histograms show the relationship between the Standard and the LessBias central, cortical GM R_1-values. The red lines indicate the perfect relationship between the data and the dashed, black lines indicate the best linear fit with an intercept of zero.

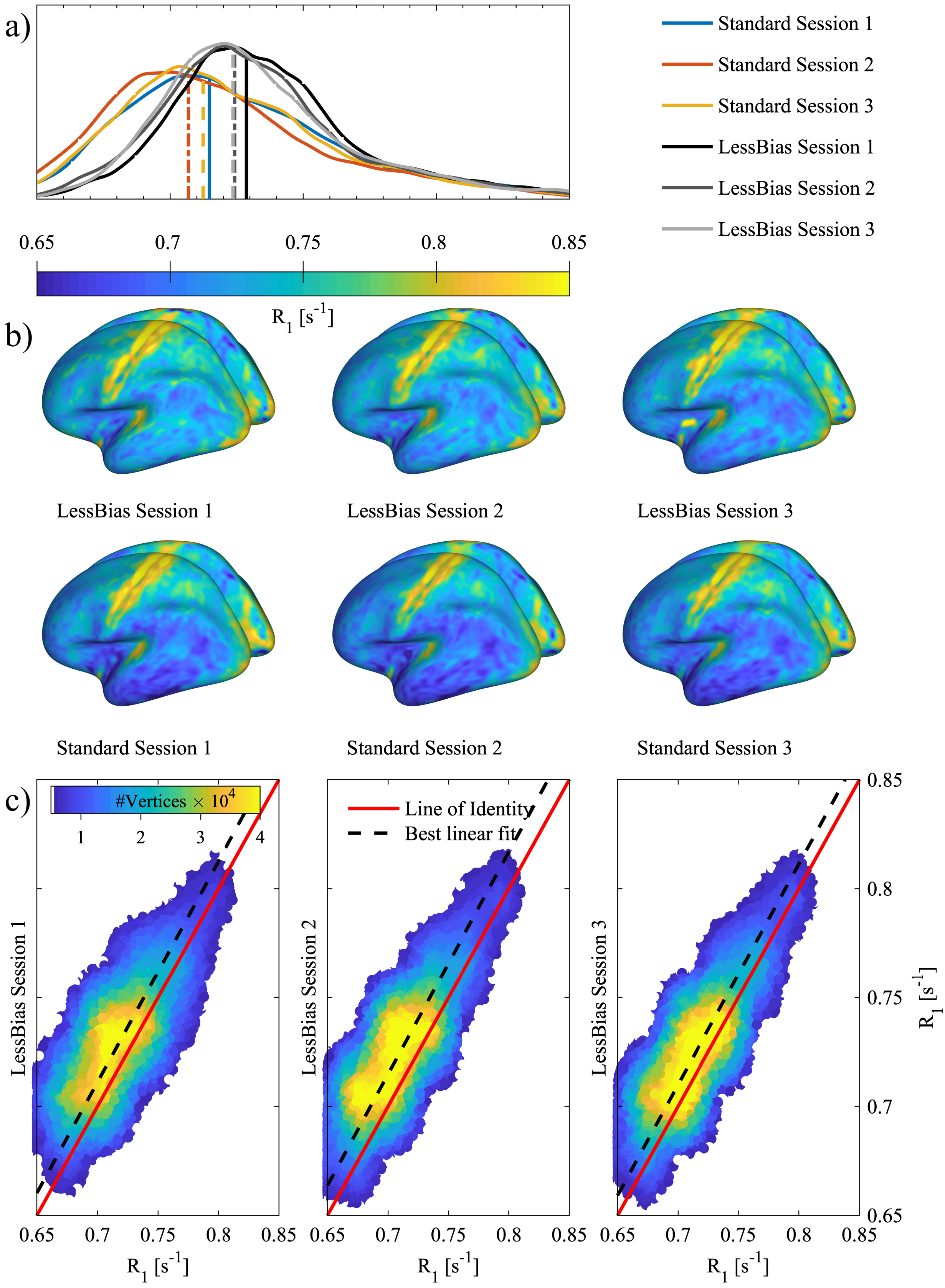

Figure S6: GM R_1-assessment, Subject 2 Session 1-3, regarding multiple sessions. Panel a) shows the distribution of R_1-values on the central GM-surface for both Standard and LessBias across three scanning sessions. Panel b) shows the surfaces corresponding to the curves in panel a). Panel c) shows 2D histograms of the relationship between Standard and LessBias central GM cortical R_1-values. It is seen that both Standard and LessBias are quite consistent across sessions, although not quite at the level seen in subject1 Figure S5 where a less motion was present. See Table S5 for a whole brain comparison.

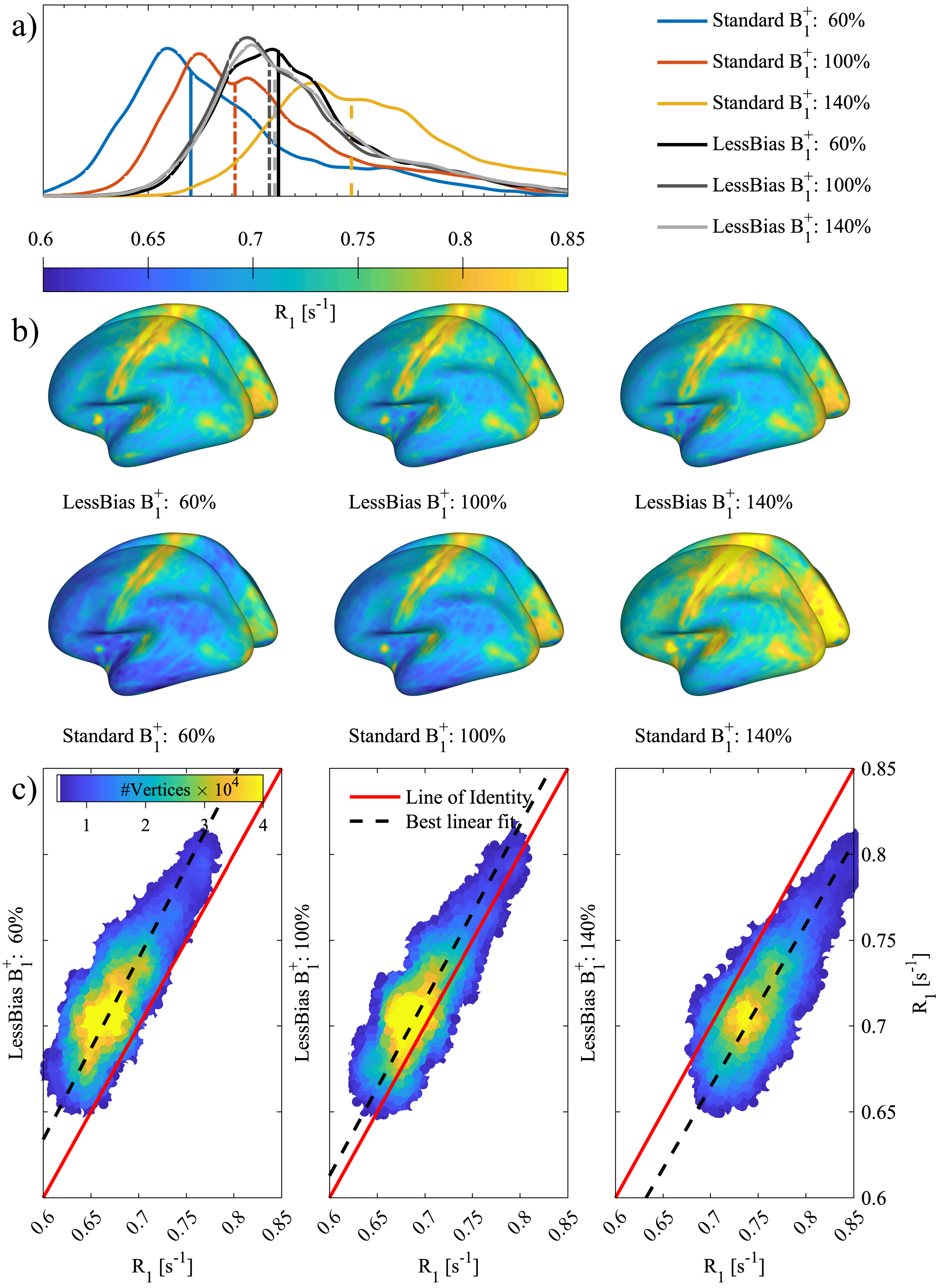

Figure S7: GM R_1-assessment, Subject 3 Session 1, regarding multiple B_1^+-values. Panel a) shows distributions of R_1-values on the central GM-surface for both Standard and LessBias, across three B_1^+-manipulations. From the distributions and their median values (indicated with vertical lines) it is clear that LessBias method is quite consistent across B_1^+-values, but Standard fluctuates rapidly. Panel b) shows the surfaces corresponding to the curves in panel a). The Standard also visually shows a substantial fluctuation in R_1-values, whereas LessBias shows much less variation. Panel c) shows 2D histograms of the relationship between Standard and LessBias central GM cortical R_1-values. It is overall seen that LessBias presents with more constant distributions across the B_1^+-manipulations than Standard, and that Standard agrees most with LessBias when B_1^+: 100 %.

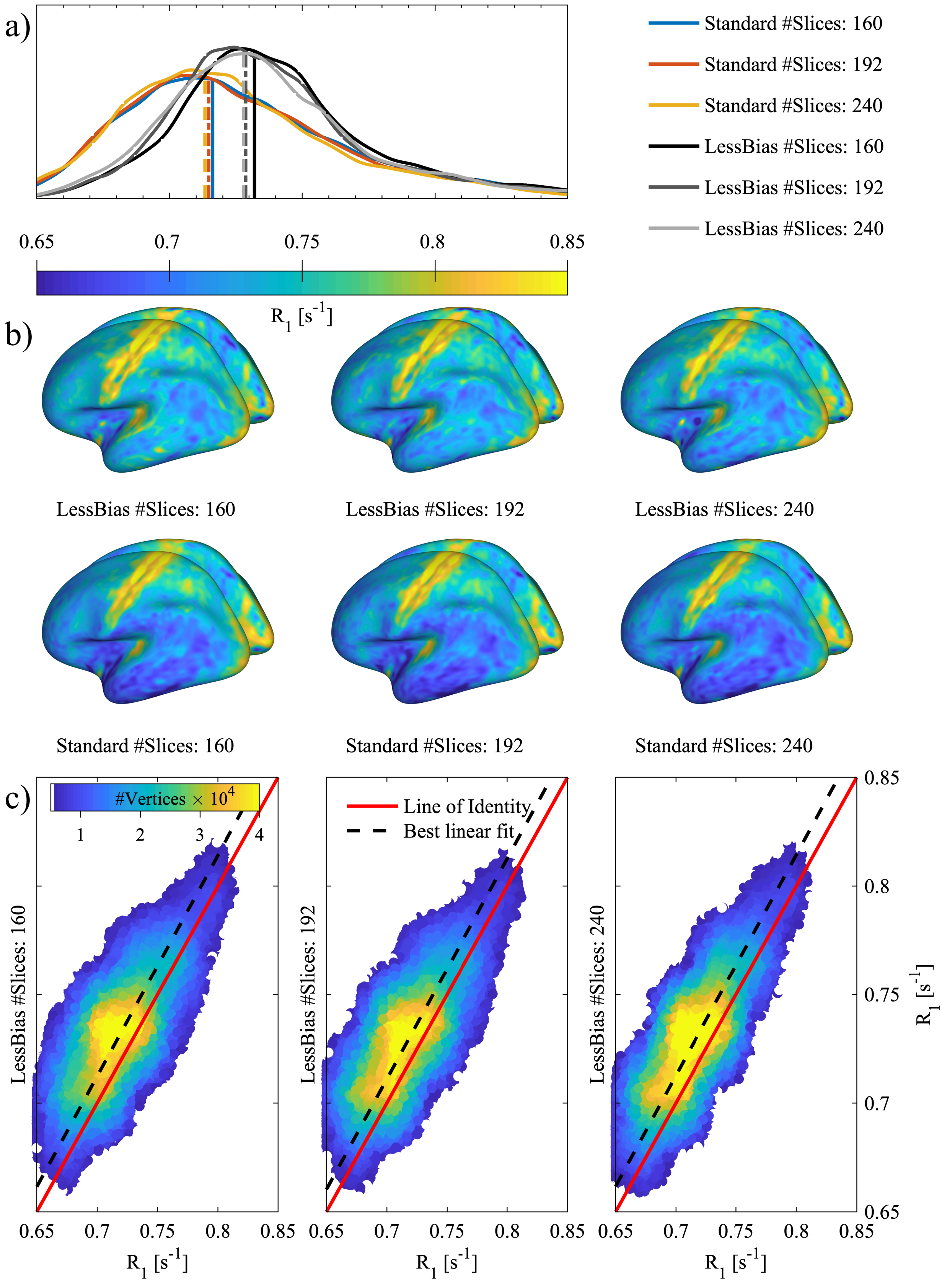

Figure S8: GM R_1-assessment, Subject 2 Session 1, regarding number of slices. Panel a) shows distributions of R_1-values on the central GM-surface for both Standard and LessBias for the different number of slices. Panel b) shows the surfaces corresponding to the curves in panel a). Panel c) shows 2D histograms of the relationship between Standard and LessBias central GM cortical R_1-values. It is seen that in the examined interval, both protocols seem quite robust to changes in the number of slices. See Table S7 for a whole brain comparison.

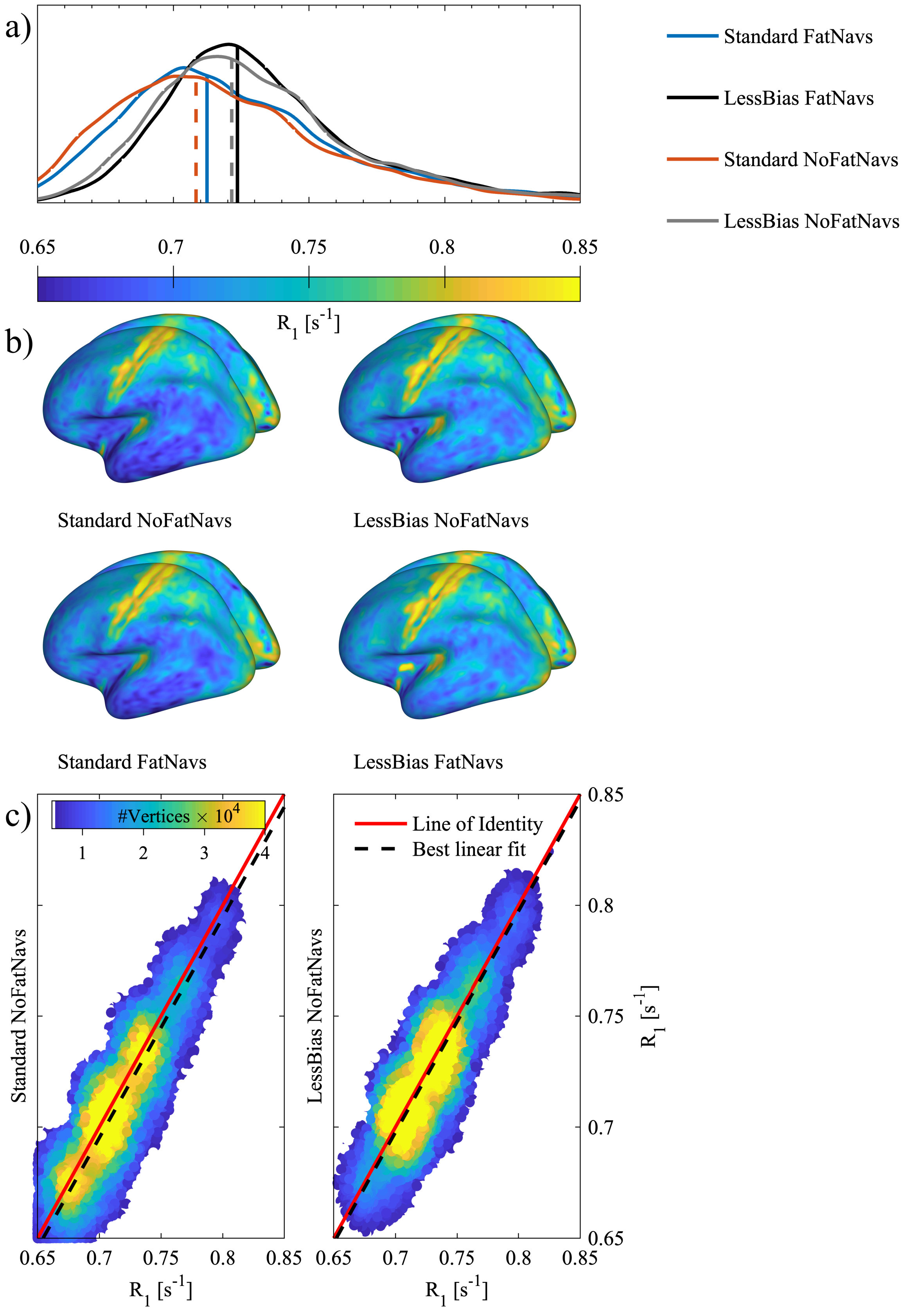

Figure S9: GM R_1-assessment, Subject 2 Session 3, regarding the fat navigator module. Panel a) shows distributions of R_1-values on the central GM-surface for both Standard and LessBias, with and without a fat navigator module present in the sequence. To be clear, "FatNavs" indicates that the acquisition was performed using the FatNavs-MP2RAGE sequence (Gallichan et al. MRM 75 (2016) 1030-1039), whereas "NoFatNavs" indicates that the MP2RAGE product version without fat navigators was used. The data in this analysis from the "FatNavs" scans, however, was the non-motion corrected data set. From the distributions (median values indicated with vertical lines) it is clear that the fat navigator pulse have no effect by itself. Panel b) shows the surfaces corresponding to the curves in panel a). In panel c), 2D histograms also show the lack of effect from the fat navigator module. The red lines indicate the perfect relationship between the data and the dashed, black lines indicate the best linear fit with an intercept of zero. An almost perfect correlation between the two MP2RAGE protocols is seen for both LessBias and Standard, indicating that the FatNavs pulse did not, by it self, influence the estimated R_1-values.

7 Effect of B_1^+-correction.

While the un-manipulated R_1-values from Standard and LessBias are fairly similar, there are reasons to believe that the un-manipulated Standard R_1-map is already affected by B_1^+-inhomogeneity. To compare our proposed method to a B_1^+-map-based correction method, we followed the SA2RAGE B_1^+-correction method (Eggenschwiler et al. MRM 67 (2012) 1609-1619) and acquired such scans accordingly. However, as the genuine SA2RAGE sequence was not available to us at time for this study, we used our MP2RAGE product sequence and switched the MP pulse to saturation mode instead of inversion. As a consequence hereof we do not directly get the real-value-ratio image, which was used for the B_1^+-mapping by Eggenschwiler et al. To accommodate this challenge we reconstructed the raw data to both produce an image containing the ratio between the real values of the two saturation images, but also a unified image, as outputted by the modified MP2RAGE sequence on the scanner. By modifying the equation behind the SA2RAGE LUT, we could convert both unified RAGE images and real-value-ratio images into B_1^+-maps. As seen in see Figure S10, we found the modified approach substantially more stable, and hence we used unified RAGE images for our in-vivo B_1^+-corrections.

In order to be able to perform a B_1^+-correction of protocols with a non-bijective transfer curve, we developed a 3D-LUT B_1^+-correction, which can work on I_{UNI}, I_{DSR} and B_1^+-images simultaneously. In Figure S11 to Figure S15, we show the impact of B_1^+-correction of the images from the Standard protocol. For these corrections we used a 3D-LUT weighting of 100:0:100 thereby ignoring the information in I_{DSR} for the Standard protocol. As an example of a true 3D-LUT B_1^+-correction, we show in Figure S16 the result of B_1^+-correction of a LessBias acquisition with 240 slices. In this case, we used a weighting of 100:1:100 to also incorporate the information found in I_{DSR}. Even with a protocol slightly more sensitive to B_1^+-inhomogenieties than the one acquired with 192 slices, we see almost no effect of the B_1^+-correction of the images from the LessBias acquisition.

Of note is that the same principles by which the 3D-LUT is performed, can trivially be extended to an ND-LUT application. Additional information, like M_0-maps, may be provided for further corrections, at the cost of computation time. We speculate that it may further be possible to filter out and estimate maps for one type of artefact outside of the provided information, by using the distance metric provided by the ND-LUT to determine the optimal solution across the estimated value, but this is subject of future investigation.

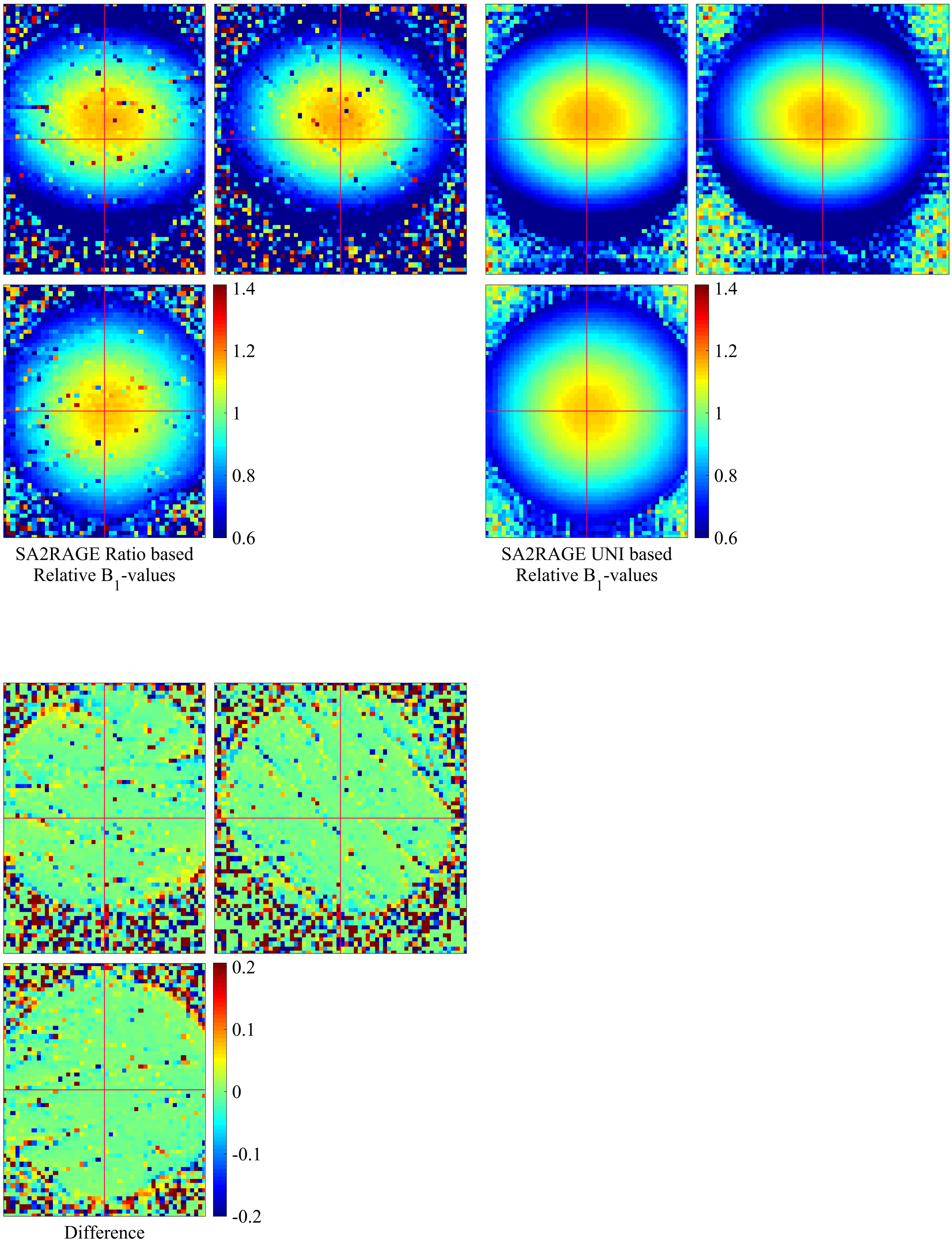

Figure S10: Phantom B_1^+-maps. Instead of the original, ratio-based SA2RAGE method (three, top left images), we estimated our B_1^+-maps (three, top right images) through the I_{UNI}-values and performed the correct via a 3D-LUT procedure. The essential difference between the two procedures is shown in the three bottom left images.

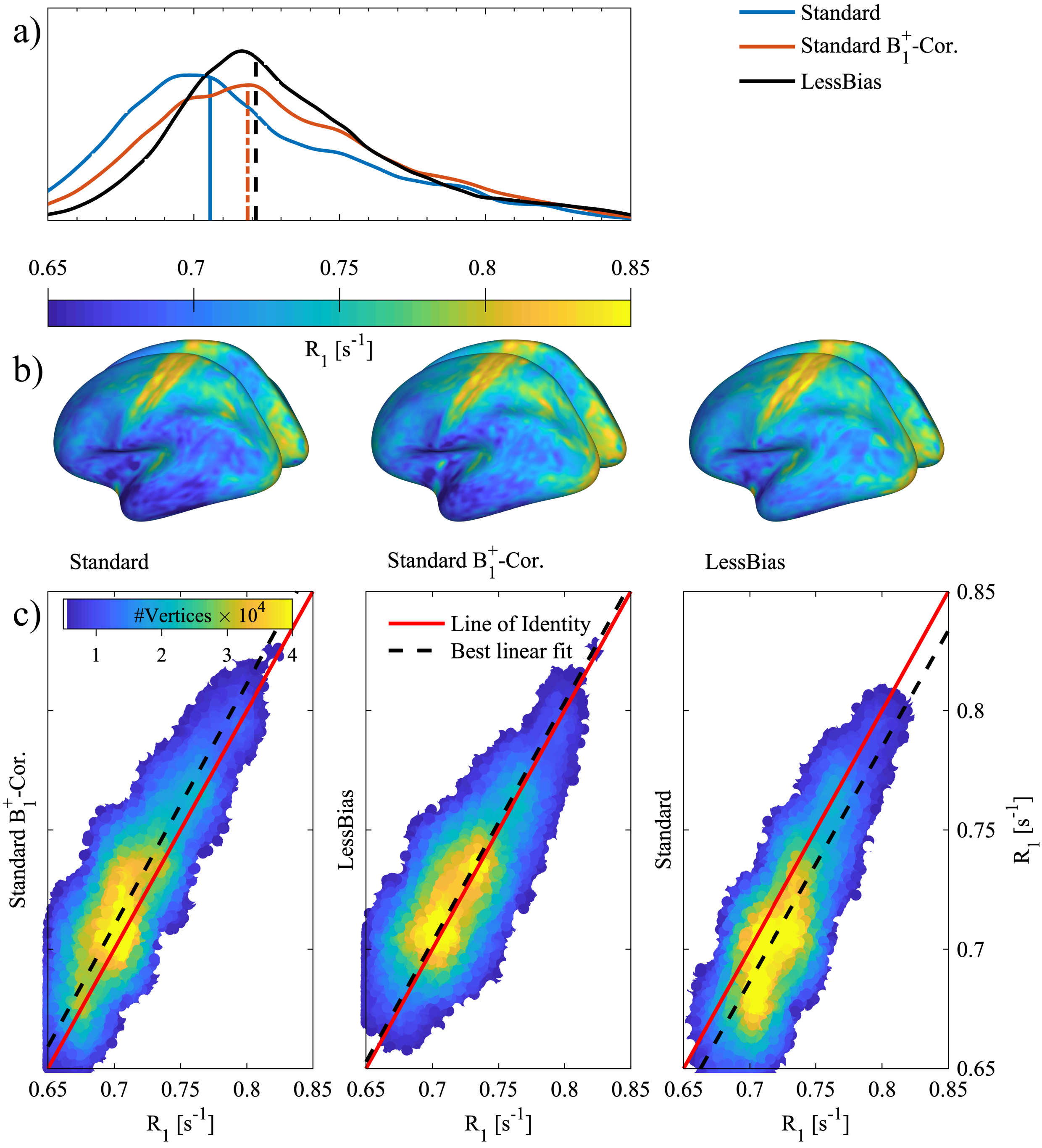

Figure S11: GM R_1-assessment, Subject 1 Session 2, regarding B_1^+-correction. Panel a) shows the distribution of R_1-values on the central GM-surface for both Standard and LessBias, As well as for a B_1^+-corrected version of Standard. From the distributions and their median values (indicated with vertical lines) it is clear that the R_1-values from Standard resemble those of LessBias, when the R_1-map from Standard is B_1^+-corrected. Panel b) shows the surfaces corresponding to the curves in panel a). A shift towards higher R_1-values at the cortex is seen, when the R_1-map from Standard is B_1^+-corrected. Panel c) shows 2D histograms for the three possible combinations of the maps in panel b). The red lines indicate the perfect relationship between the data and the dashed, black lines indicate the best linear fit with an intercept of zero. It is seen from the plots that while the maps from LessBias and Standard are fairly similar, the best correspondence is found between the R_1-maps from B_1^+-corrected Standard and LessBias.

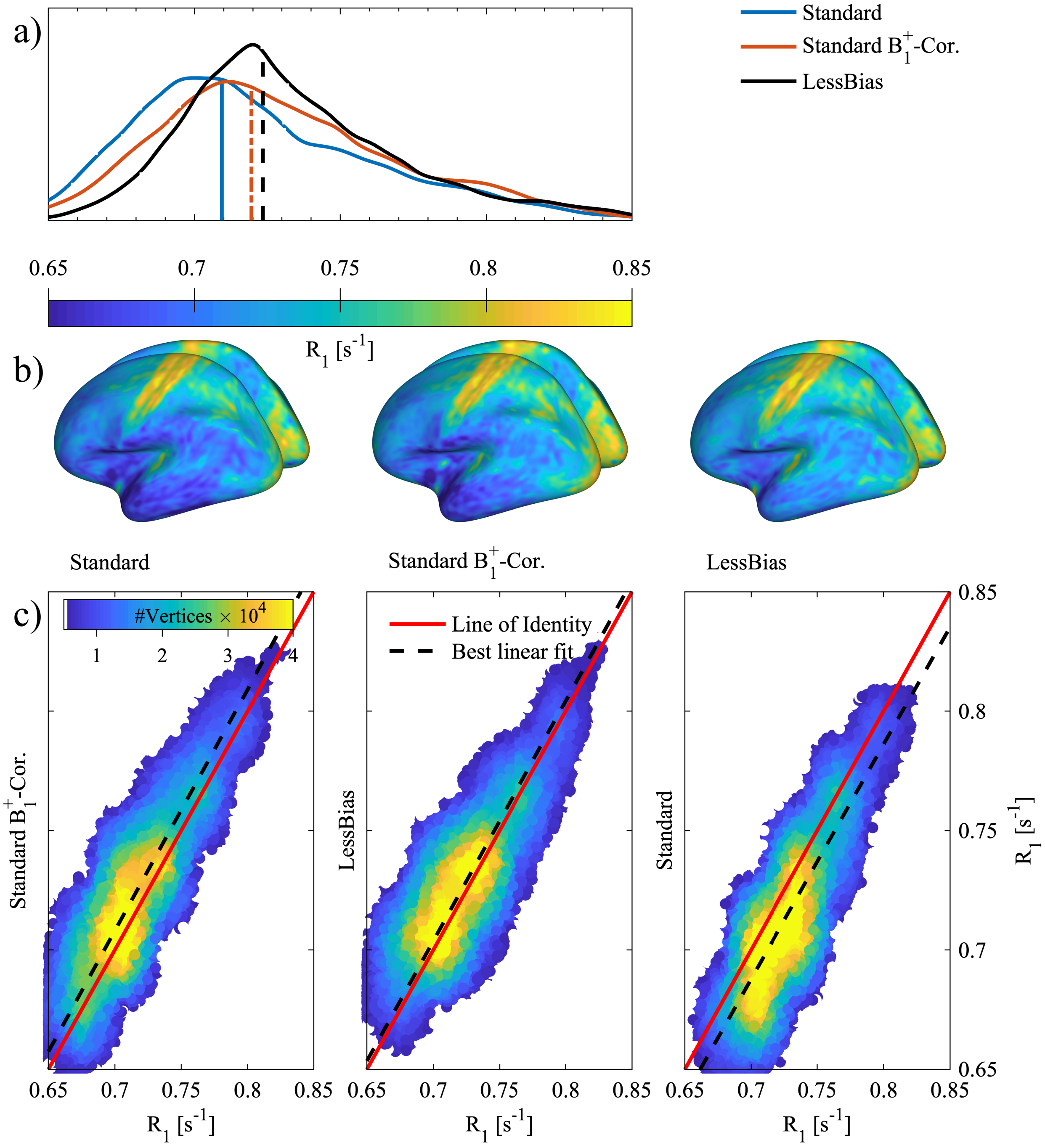

Figure S12: GM R_1-assessment, Subject 1 Session 3, regarding B_1^+-correction. Panel a) shows the distribution of R_1-values on the central GM-surface for both Standard and LessBias, As well as for a B_1^+-corrected version of Standard. From the distributions and their median values (indicated with vertical lines) it is clear that the R_1-values from Standard resemble those of LessBias, when the R_1-map from Standard is B_1^+-corrected. Panel b) shows the surfaces corresponding to the curves in panel a). A shift towards higher R_1-values at the cortex is seen, when the R_1-map from Standard is B_1^+-corrected. Panel c) shows 2D histograms for the three possible combinations of the maps in panel b). The red lines indicate the perfect relationship between the data and the dashed, black lines indicate the best linear fit with an intercept of zero. It is seen from the plots that while the maps from LessBias and Standard are fairly similar, the best correspondence is found between the R_1-maps from B_1^+-corrected Standard and LessBias.

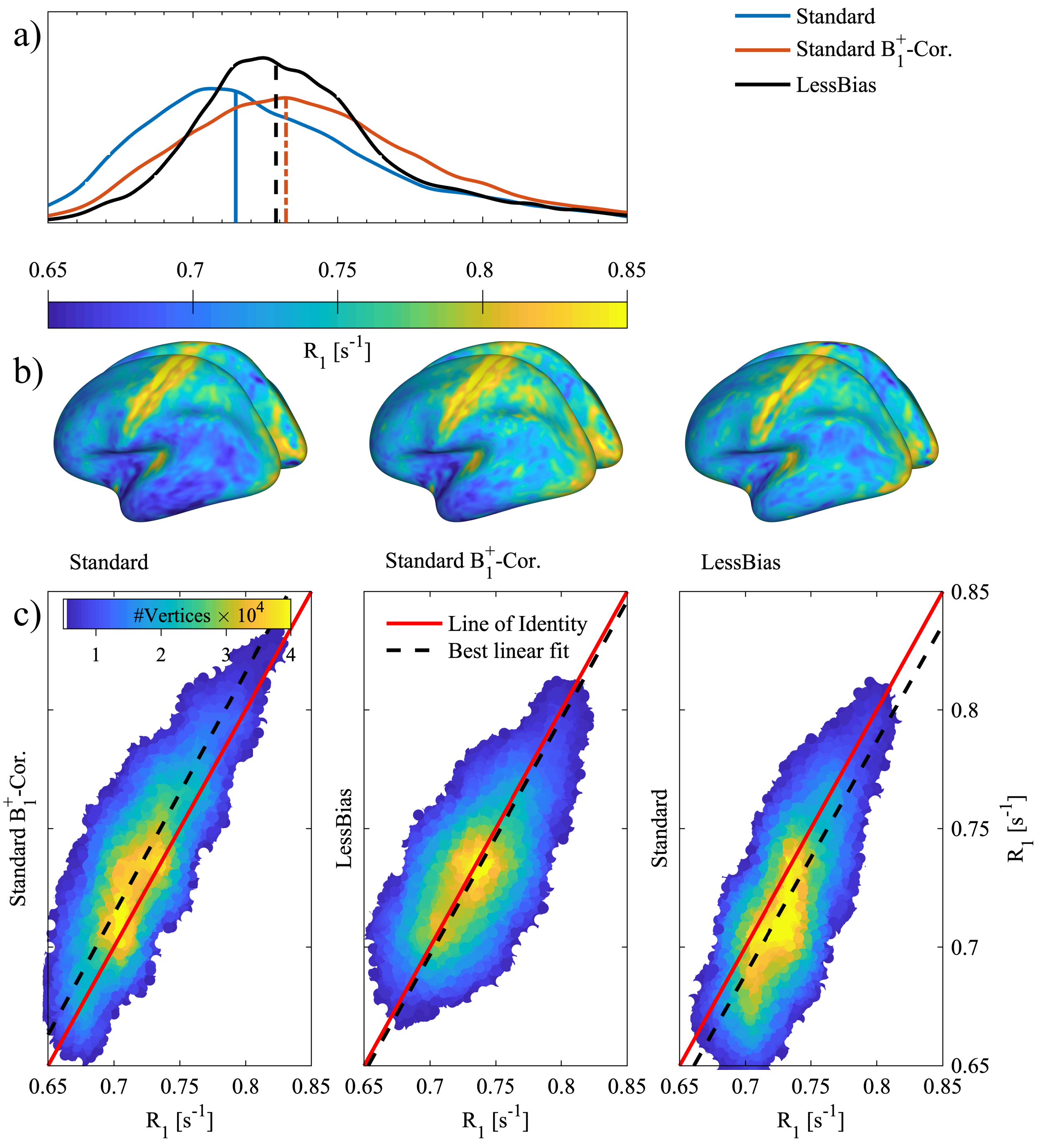

Figure S13: GM R_1-assessment, Subject 2 Session 1, regarding B_1^+-correction. Panel a) shows the distribution of R_1-values on the central GM-surface for both Standard and LessBias, As well as for a B_1^+-corrected version of Standard. From the distributions and their median values (indicated with vertical lines) it is clear that the R_1-values from Standard resemble those of LessBias, when the R_1-map from Standard is B_1^+-corrected. Panel b) shows the surfaces corresponding to the curves in panel a). A shift towards higher R_1-values at the cortex is seen, when the R_1-map from Standard is B_1^+-corrected. Panel c) shows 2D histograms for the three possible combinations of the maps in panel b). The red lines indicate the perfect relationship between the data and the dashed, black lines indicate the best linear fit with an intercept of zero. It is seen from the plots that while the maps from LessBias and Standard are fairly similar, the best correspondence is found between the R_1-maps from B_1^+-corrected Standard and LessBias.

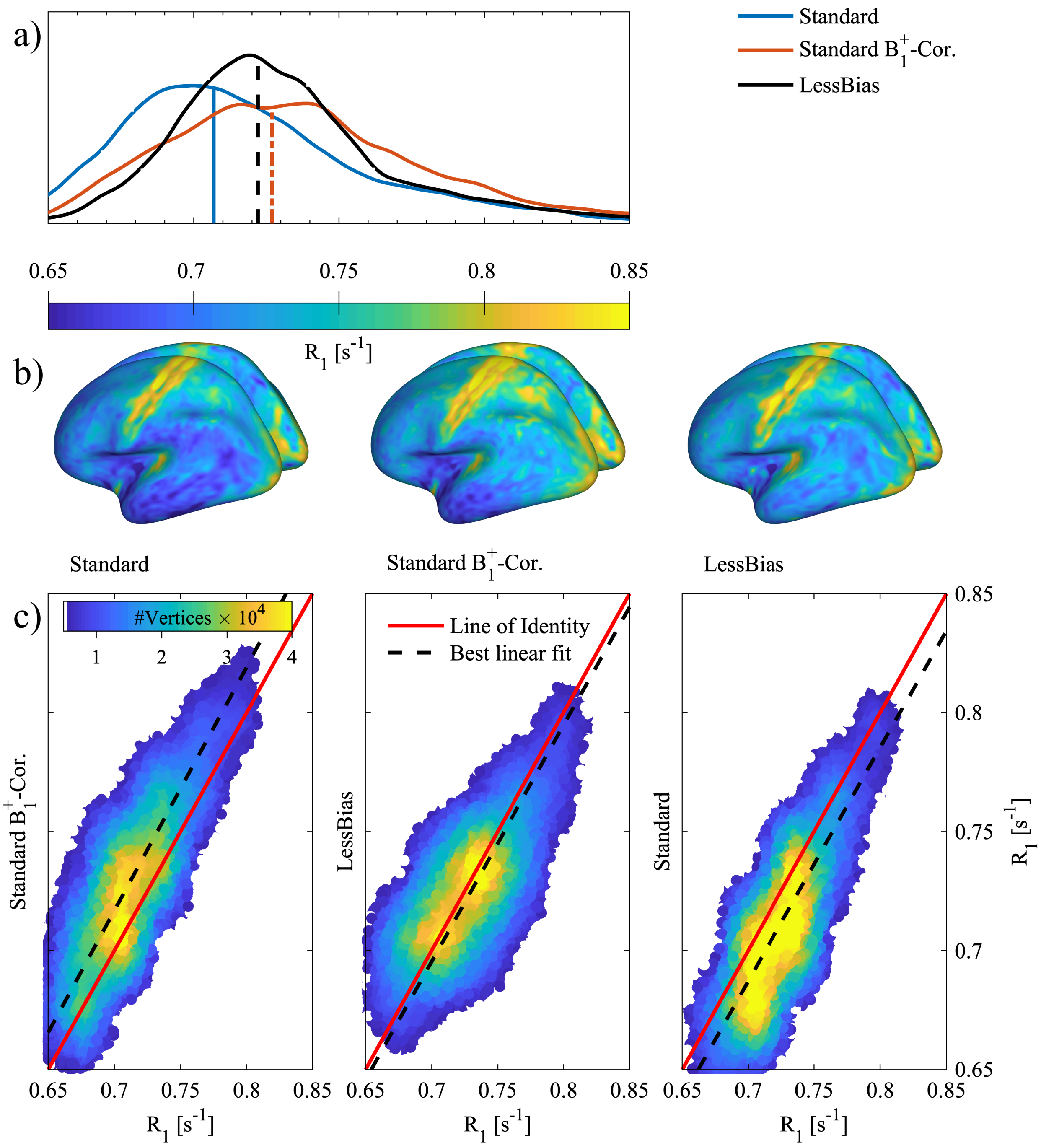

Figure S14: GM R_1-assessment, Subject 2 Session 2, regarding B_1^+-correction. Panel a) shows the distribution of R_1-values on the central GM-surface for both Standard and LessBias, As well as for a B_1^+-corrected version of Standard. From the distributions and their median values (indicated with vertical lines) it is clear that the R_1-values from Standard resemble those of LessBias, when the R_1-map from Standard is B_1^+-corrected. Panel b) shows the surfaces corresponding to the curves in panel a). A shift towards higher R_1-values at the cortex is seen, when the R_1-map from Standard is B_1^+-corrected. Panel c) shows 2D histograms for the three possible combinations of the maps in panel b). The red lines indicate the perfect relationship between the data and the dashed, black lines indicate the best linear fit with an intercept of zero. It is seen from the plots that while the maps from LessBias and Standard are fairly similar, the best correspondence is found between the R_1-maps from B_1^+-corrected Standard and LessBias.

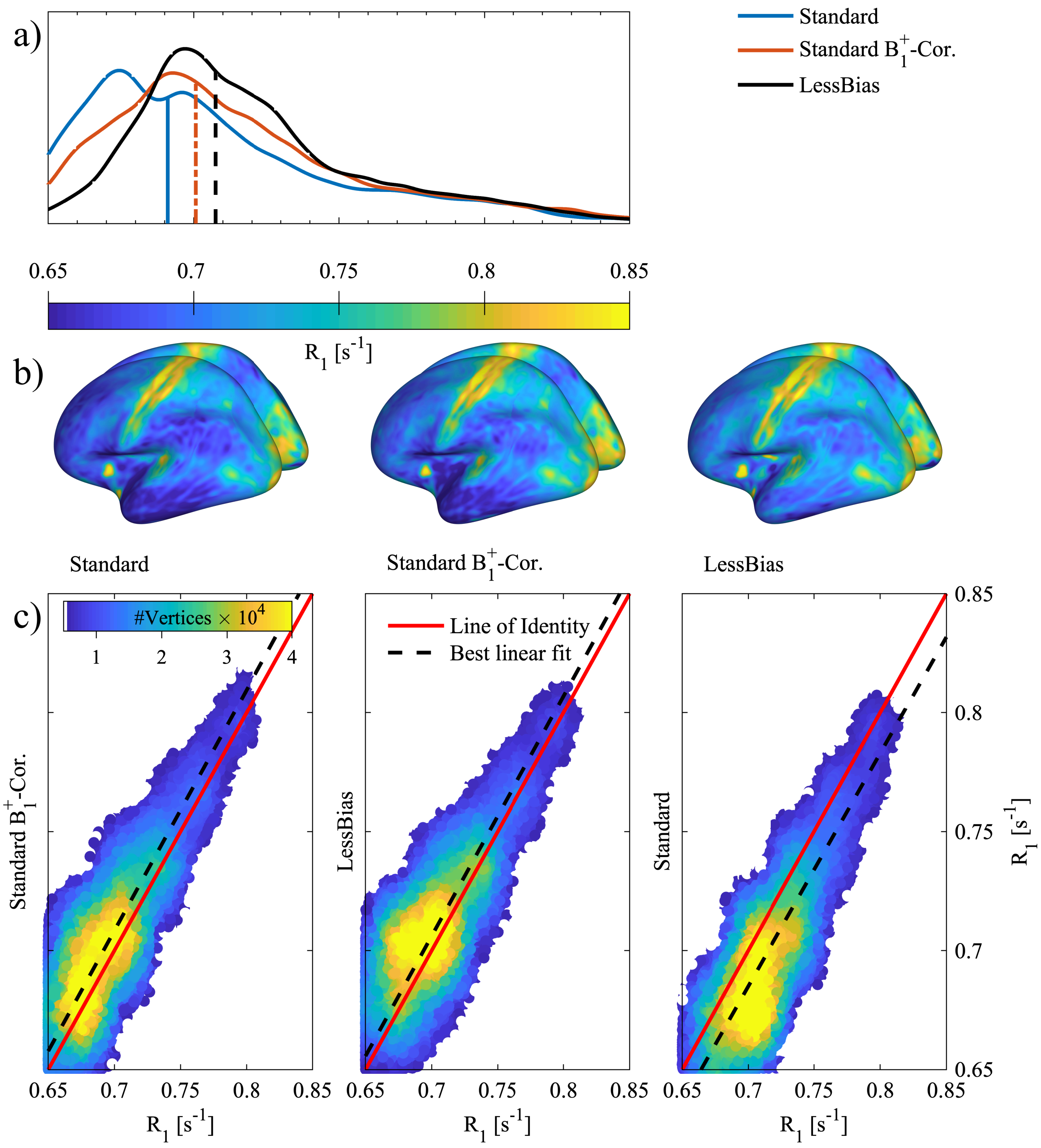

Figure S15: GM R_1-assessment, Subject 3 Session 1, regarding B_1^+-correction. Panel a) shows the distribution of R_1-values on the central GM-surface for both Standard and LessBias, As well as for a B_1^+-corrected version of Standard. From the distributions and their median values (indicated with vertical lines) it is clear that the R_1-values from Standard resemble those of LessBias, when the R_1-map from Standard is B_1^+-corrected. Panel b) shows the surfaces corresponding to the curves in panel a). A shift towards higher R_1-values at the cortex is seen, when the R_1-map from Standard is B_1^+-corrected. Panel c) shows 2D histograms for the three possible combinations of the maps in panel b). The red lines indicate the perfect relationship between the data and the dashed, black lines indicate the best linear fit with an intercept of zero. It is seen from the plots that while the maps from LessBias and Standard are fairly similar, the best correspondence is found between the R_1-maps from B_1^+-corrected Standard and LessBias.

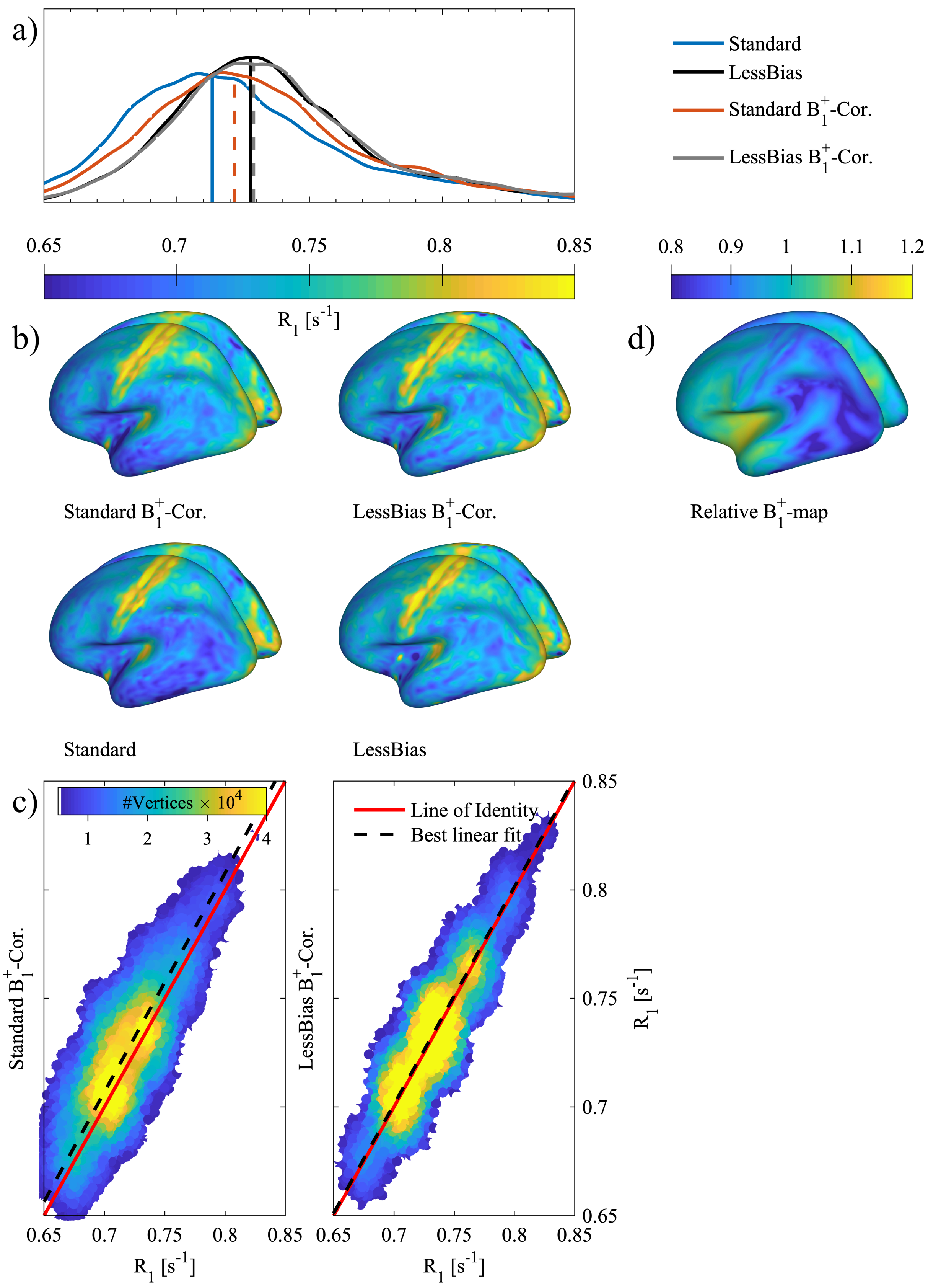

Figure S16: GM R_1-assessment, Subject 2 Session 1, regarding B_1^+-correction. Panel a) shows distributions of R_1-values on the central GM-surface for both protocols, with and without B_1^+-correction. From the distributions and their median values (indicated with vertical lines) it is clear that the R_1-values from Standard resemble those of LessBias, when the R_1-map from Standard is B_1^+-corrected. However, as could be expected from Figure 5, the effect of B_1^+-correction on LessBias is minimal. Panel b) shows the surfaces corresponding to the curves in panel a). A shift towards higher R_1-values at the cortex is seen, when the R_1-map from Standard is B_1^+-corrected. In panel c), 2D histograms show the effect of B_1^+-correction for the two different protocols. The red lines indicate the perfect relationship between the data and the dashed, black lines indicate the best linear fit with an intercept of zero. It is seen that LessBias and LessBias with B_1^+-correction have a stronger correlation than Standard and Standard with B_1^+-correction. Panel d) shows the B_1^+-map used for the correction.

8 A 7T example with 512 slices

As the desired resolution increases, a larger number of slices is required. Unfortunately this increases the B_1^+-inhomogeneity sensitivity, which counteracts the resolution gains if left untreated. Some of this inhomogeneity sensitivity can be counteracted by longer $T_{\mathrm{R,MP2RAGE}}$ and shorter echo spacing, i.e. $T_{\mathrm{R,FLASH}}$.

Additionally, pTx, e.g., with universal pulses, can be used to reduce the severity of the B_1^+-inhomogeneity effect, typically to a near 3T like scenario, with a relative $B_1^+ = \pm 20\%$. In Figure S17, we show the transfer function for a Standard* and a LessBias* protocol which have been modified to include 512 slices. Both protocols use $T_{\mathrm{R,MP2RAGE}} = 6\,\mathrm{s}$ and $T_{\mathrm{R,FLASH}} = 3.9\,\mathrm{ms}$. Other parameters were; Standard*: $\mathrm{FA}_1/\mathrm{FA}_2 = 4°/5°$, $T_{\mathrm{I},1}/T_{\mathrm{I},2} = 700\,\mathrm{ms}/2500\,\mathrm{ms}$, LessBias*: $\mathrm{FA}_1/\mathrm{FA}_2 = 3°/5°$, $T_{\mathrm{I},1}/T_{\mathrm{I},2} = 500\,\mathrm{ms}/2000\,\mathrm{ms}$. Similar to our 3T example, Figure S17 panel **a** shows that, with a bijective transfer function, a protocol with 512 slices will show substantial B_1^+-inhomogeneity sensitivity compared to a protocol with a non-bijective transfer function (Figure S17 panel **b**). One thing to note in Figure S17 panel **b**, is the steeper (negative) slope around the T_1 of GM, caused by the higher T_1 at 7T compared to 3T. This could lead to slightly increased noise levels in GM, but can be counteracted by an increased $T_{\mathrm{R,MP2RAGE}}$, $T_{\mathrm{I},1}$ and $T_{\mathrm{I},2}$ if deemed problematic.

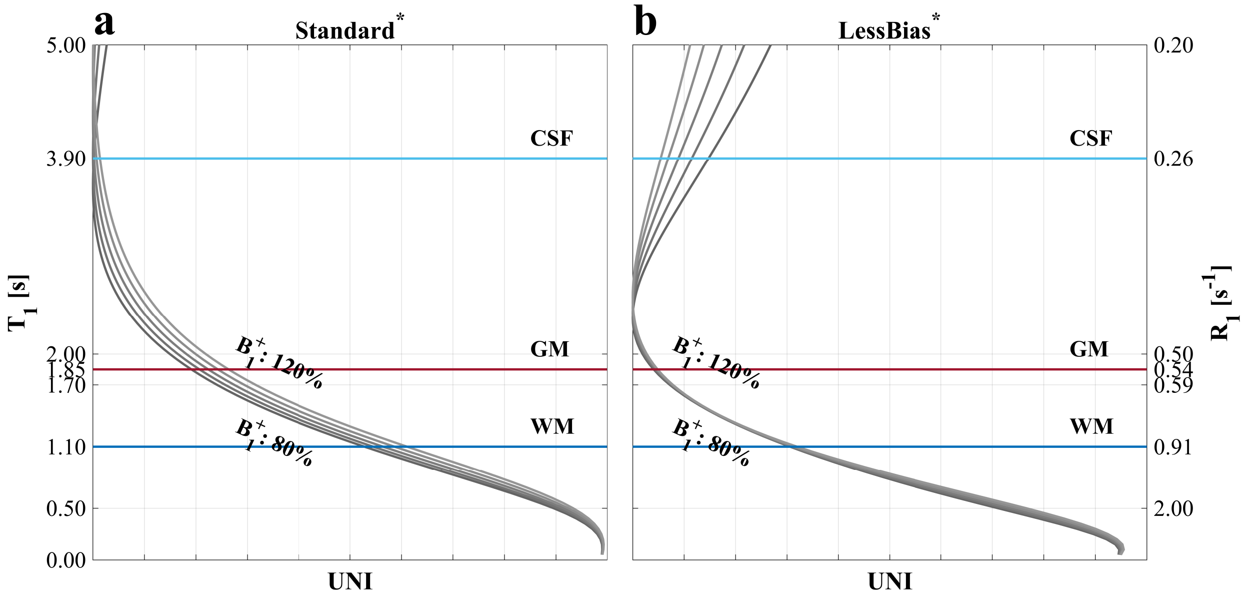

Figure S17: Transfer functions for two protocols **a**: Standard* and **b**: LessBias* modified to acquire 512 slices at 7T.
